# Sex-related differences in self-reported treatment burden in patients with atrial fibrillation

**DOI:** 10.3389/fcvm.2022.1029730

**Published:** 2022-11-04

**Authors:** Miroslav Mihajlovic, Jelena Simic, Milan Marinkovic, Vladan Kovacevic, Aleksandar Kocijancic, Nebojsa Mujovic, Tatjana S. Potpara

**Affiliations:** ^1^School of Medicine, University of Belgrade, Belgrade, Serbia; ^2^Cardiology Clinic, University Clinical Centre of Serbia, Belgrade, Serbia

**Keywords:** atrial fibrillation, treatment burden, sex-related differences, quality of life, female sex

## Abstract

**Background:**

Treatment burden (TB) is defined as the patient’s workload of healthcare and its impact on patient functioning and wellbeing. High TB can lead to non-adherence, a higher risk of adverse outcomes and lower quality of life (QoL). We have previously reported a higher TB in patients with atrial fibrillation (AF) vs. those with other chronic conditions. In this analysis, we explored sex-related differences in self-reported TB in AF patients.

**Materials and methods:**

A single-center, prospective study included consecutive patients with AF under drug treatment for at least 6 months before enrollment from April to June 2019. Patients were asked to voluntarily and anonymously answer the Treatment Burden Questionnaire (TBQ). All patients signed the written consent for participation.

**Results:**

Of 331 patients (mean age 65.4 ± 10.3 years, mean total AF history 6.41 ± 6.62 years), 127 (38.4%) were females. The mean TB was significantly higher in females compared to males (53.7 vs. 42.6 out of 170 points, *p* < 0.001), and females more frequently reported TB ≥ 59 points than males (37.8% vs. 20.6%, *p* = 0.001). In females, on multivariable analysis of the highest TB quartile (TB ≥ 59), non-vitamin K Antagonist Oral Anticoagulant (NOAC) use [Odds Ratio (OR) 0.319; 95% Confidence Interval (CI) 0.12–0.83, *P* = 0.019], while in males, catheter ablation and/or ECV of AF (OR 0.383; 95% CI 0.18–0.81, *P* = 0.012) were negatively associated with the highest TB quartile.

**Conclusion:**

Our study was the first to explore the sex-specific determinants of TB in AF patients. Females had significantly higher TB compared with males. Approximately 2 in 5 females and 1 in 5 males reported TB ≥ 59 points, previously shown to be an unacceptable burden of treatment for patients. Using a NOAC rather than vitamin K antagonist (VKA) in females and a rhythm control strategy in males could decrease TB to acceptable values.

## Introduction

Treatment burden (TB) is defined as the workload of healthcare (including time invested by a patient due to treatment and self-monitoring of chronic health conditions) and its impact on patient functioning and wellbeing (1). Patient capacity to endure treatment workload varies and depends on a variety of psychological, physical, and social factors (2, 3). One research showed that patients with three chronic health conditions spent a mean of 49.6–71.0 h on health-related activities and 1–6 visits to a healthcare giver per month, and a patient’s workload increased with an increasing number of chronic health conditions (4). High TB can lead to patient non-adherence (5–7), exacerbation of chronic health conditions, higher hospitalization rate, and higher mortality, as well as lower quality of life (QoL) (8–10).

Treatment of chronic health conditions is time-consuming for both patients and physicians (11). The new concept of a minimally disruptive medicine approach aims to achieve the patient’s goals in health and life while minimizing the patient’s workload and suggests that care should move from the disease-centered to more patient-centered models of care (11, 12).

Several disease-specific and general tools have been developed and validated for the assessment of TB (13). Recently, a study in France (2,413 patients with one or more chronic conditions) reported a TBQ score cutoff of 59 points (out of 150 points) to be an unacceptably high burden of treatment for patients (14). In our main study, we assessed TB in patients with AF and found that approximately 1 in 4 patients with AF has TB ≥ 59 points; among others, the female sex was reported as an independent predictor of a TBQ score value of ≥59 points (15). Also, other studies showed that the female sex was associated with higher TB in patients with various chronic health conditions (16–18), but to our knowledge, there are no studies that address the sex differences in self-reported TB in patients with atrial fibrillation (AF).

In the present analysis, we explore sex differences in self-reported TB and the association of quality of life with TB in patients with AF.

## Materials and methods

### Study population

The detailed study protocol has been previously reported (15). Briefly, 514 consecutive adult in- or outpatients seen in the University Clinical Centre of Serbia from April to June 2019, who has been under treatment for at least 6 months before enrollment, were invited to voluntarily and anonymously answer the study questionnaires. Patients prescribed therapy within less than 6 months were excluded from the study to avoid under- or overestimation of the TB.

**FIGURE 1:**
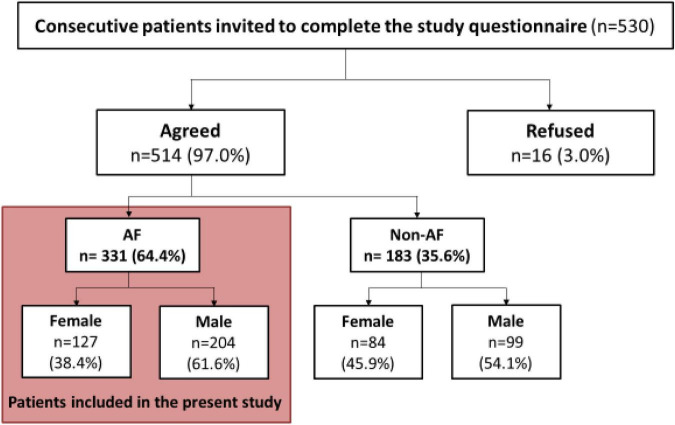
Study flowchart. AF, atrial fibrillation.

This exploratory analysis included the subset of patients with AF (*n* = 331) from the main study cohort, see Figure 1 (15).

The study questionnaires included the modified Treatment Burden Questionnaire (TBQ, see Supplementary Table 1) and the EQ-5D questionnaire assessing QoL. The modification of TBQ in our study refers to the first four questions of the original TBQ addressing several aspects of taking medications, where we separately asked questions for oral anticoagulant therapy (OAC) and all other medication. The rationale for modifying the original TBQ was to elucidate specific treatment burden related to OAC. The remaining nine questions address the burden associated with laboratory testing, self-monitoring of health [e.g., measurements of blood glucose, blood pressure or international normalized ratio (INR)], doctor visits, administrative tasks (e.g., arranging visit appointments, health-related paperwork), the effect(s) of diet restrictions/modification and physical exercise requirements and social impact of the treatment. The financial burden associated with healthcare was excluded from the questionnaire because all Serbia citizens have a free national public health insurance program [however, specific drugs such as non-vitamin K antagonist oral anticoagulants (NOAC) are not reimbursed], which could have inflated the TB. Patient demographics, chronic health conditions (only confirmed diagnoses using International Classification of Diseases 10 were included), and current therapy were recorded by the study investigator. Multimorbidity was defined as the coexistence of two or more chronic health conditions (19) other than AF. Polypharmacy was defined as the concomitant use of five or more medications daily (20).

All patients signed the written consent for participation, and the Ethical Committee approved the study at the School of Medicine, Belgrade University.

### Statistical analysis

Patients with AF were stratified and analyzed by sex. Continuous variables were shown as mean with standard deviation (SD) or median with interquartile range (IQR), while the nominal variables were shown as frequencies and percentages. Sex-specific differences in baseline demographics, comorbid conditions, characteristics of AF, and current treatment were analyzed using the Student’s *t*-test for continuous variables or the Chi-Square test for categorical data.

Patients were instructed to grade each TB question from 1 (the lowest burden) to 10 (the highest burden) points. The total TB score value was calculated as a sum of question-specific points, and the maximum possible score value is 170 points. Therefore, an individual patient’s TB score is expressed as a sum of points and a percentage of the maximum score value.

The self-reported TB was analyzed as a continuous variable with the Linear Regression method. In addition, TB was divided into quartiles and analyzed with the Binary Logistic Regression method. We examined the relationship of baseline variables listed in Table 1 with total TB, the highest and lowest TB quartile on univariate analyses. Multivariate models with total TB and the highest/lowest TB quartile as independent variables were conducted using the statistically significant variables in univariate analyses.

**TABLE 1:** Sex differences in socio-demographic characteristics of the study cohort, concomitant comorbidity and current medication in atrial fibrillation (AF) patients.

Variable	AF patients *n* = 331 (%)	Female *n* = 127 (38.4)	Male *n* = 204 (61.6)	*P*-value
Age (mean, ±SD)	65.42 ± 10.32	67.92 ± 8.74	63.86 ± 10.92	<0.001
Age ≤ 64	133 (40.2)	36 (28.3)	97 (47.5)	<0.001
Age 65–74	141 (42.6)	63 (49.6)	78 (38.2)	0.042
Age ≥ 75	57 (17.2)	28 (22.0)	29 (14.2)	0.074
***Education degree***				
Elementary	44 (13.3)	24 (18.9)	20 (9.8)	0.020
High school	165 (49.8)	63 (49.6)	102 (50.0)	1.000
College	47 (14.2)	18 (14.2)	29 (14.2)	1.000
University	75 (22.7)	22 (17.3)	53 (26.0)	0.079
***Employment status***				
Employed	83 (25.1)	14 (11.0)	69 (33.8)	<0.001
Unemployed	32 (9.7)	10 (7.9)	13 (6.4)	0.659
Retiree	49 (14.8)	103 (81.1)	122 (59.8)	<0.001
***Marital status***				
Married/living with a partner	250 (75.5)	85 (66.9)	165 (80.9)	0.006
Alone/divorced	32 (9.7)	9 (7.1)	23 (11.3)	0.253
Widow(er)	49 (14.8)	33 (26.0)	16 (7.8)	<0.001
***Cigarette smoking***				
Smoker	49 (14.8)	14 (11.0)	35 (17.2)	0.152
Former smoker	93 (28.1)	21 (16.5)	72 (35.3)	<0.001
Non-smoker	189 (57.1)	92 (72.4)	97 (47.5)	<0.001
***Functional mobility* ^1^**				
Fully mobile	313 (94.6)	118 (92.9)	195 (95.6)	0.325
Mobile with help	18 (5.4)	9 (7.1)	9 (4.4)	0.325
***Comorbid conditions***				
Hypertension	271 (81.9)	109 (85.8)	162 (79.4)	0.186
Heart failure	34 (10.3)	9 (7.1)	25 (12.3)	0.142
LVEF < 50%	50 (15.1)	6 (4.7)	44 (21.6)	<0.001
Ischemic heart disease	58 (17.5)	15 (11.8)	43 (21.1)	0.037
ACS	2 (0.6)	1 (0.8)	1 (0.5)	1.000
Prior MI	29 (8.8)	3 (2.4)	26 (12.7)	0.001
CAD	16 (4.8)	7 (5.5)	9 (4.4)	0.793
PCI/ballon angioplasty	30 (9.1)	6 (4.7)	24 (11.8)	0.031
CABG	8 (2.4)	0 (0.0)	8 (3.9)	0.026
Cardiomyopathy	30 (9.1)	7 (5.5)	23 (11.3)	0.080
Valvular disease	25 (7.6)	16 (12.6)	9 (4.4)	0.009
Supraventricular arrhythmias (SA + AFL)	52 (15.7)	18 (14.2)	34 (16.7)	0.642
Ventricular arrhythmias	30 (9.1)	9 (7.1)	21 (10.3)	0.431
CIEDs^2^	25 (7.6)	9 (7.1)	16 (7.8)	1.000
Peripheral artery disease	4 (4.1)	2 (1.6)	2 (1.0)	0.639
Diabetes mellitus type II	64 (19.3)	27 (21.3)	37 (18.1)	0.479
Prior stroke/TIA	13 (3.9)	7 (5.5)	6 (2.9)	0.257
Chronic kidney disease	28 (8.5)	8 (6.3)	20 (9.8)	0.314
COPD	24 (7.3)	9 (7.1)	15 (7.4)	1.000
Malignancy	24 (7.3)	7 (5.5)	10 (4.9)	0.803
Thyroid disfunction^3^	70 (21.2)	32 (26.0)	37 (18.1)	0.098
Hyperlipoproteinemia	114 (34.4)	43 (33.9)	71 (34.8)	0.906
Other diseases	46 (13.9)	17 (13.4)	29 (14.2)	0.872
CHA_2_DS_2_-VASc score (mean; range 0–7)	2.63 ± 1.50	3.38 ± 1.25	2.16 ± 1.45	<0.001
>1 non-sex related CHA_2_DS_2_-VASc risk factors	300 (90.6)	121 (95.3)	179 (87.7)	0.031
***AF characteristics***				
Total AF history (yrs.)	6.41 ± 6.62	5.56 ± 5.60	6.91 ± 7.13	0.074
Permanent AF	97 (29.3)	37 (29.1)	60 (29.4)	1.000
***Current medication***				
OAC	299 (90.3)	113 (89.0)	186 (91.2)	0.568
VKA	189 (57.1)	70 (55.1)	119 (58.3)	0.570
NOAC	110 (33.2)	43 (33.9)	67 (32.8)	0.905
OAC treatment duration (yrs.)	3.69 ± 3.82	3.33 ± 3.38	3.90 ± 4.05	0.224
Aspirin	35 (10.6)	14 (11.0)	21 (10.3)	0.856
P2Y12 inhibitor	27 (8.2)	8 (6.3)	19 (9.3)	0.411
Beta blocker	264 (79.8)	99 (78.0)	165 (80.9)	0.574
Non-DHP Ca blocker	8 (2.4)	3 (2.4)	5 (2.5)	1.000
Digitalis	14 (4.2)	5 (3.9)	9 (4.4)	1.000
Antiarrhythmic drugs^4^	195 (58.9)	78 (61.4)	117 (57.4)	0.492
ACEI/ARB	232 (70.1)	87 (68.5)	145 (71.1)	0.624
Diuretics	188 (70.1)	77 (60.6)	111 (54.4)	0.305
Spironolactone	81 (24.5)	25 (19.7)	56 (27.5)	0.117
Statins	135 (40.8)	57 (44.9)	78 (38.2)	0.251
Sedative	35 (10.6)	13 (10.2)	22 (10.8)	1.000
PPI	95 (28.7)	32 (25.2)	63 (30.9)	0.318
Insulin	13 (3.9)	8 (6.3)	5 (2.5)	0.089
Oral antidiabetics	50 (15.1)	22 (17.3)	28 (13.7)	0.431
Other medications	127 (38.4)	55 (43.3)	72 (35.3)	0.164
***Non-pharmacological treatment***				
Ablation/ECV	136 (41.1)	39 (30.7)	97 (47.5)	0.003
ECV AF	96 (29.0)	24 (18.9)	72 (35.3)	0.002
AF Ablation	55 (16.5)	18 (14.2)	37 (18.1)	0.367
AFL Ablation	9 (2.7)	0 (0.0)	9 (4.4)	0.014
***Multimorbidity and polypharmacy***				
Patients with polypharmacy	237 (71.6)	96 (75.6)	141 (69.1)	0.213
*N* of drugs, mean (range)	6.18 ± 2.74 (1–15)	6.37 ± 2.68	6.07 ± 2.78	0.332
*N* of pills, mean (range)	7.21 ± 3.27 (1–20)	7.37 ± 3.05	7.11 ± 3.41	0.478
*N* of drugs without OAC, mean (range)	5.24 ± 2.63 (1–14)	5.42 ± 2.58	5.13 ± 2.66	0.338
*N* of pills without OAC, mean (range)	6.31 ± 3.21 (1–19)	6.48 ± 3.03	6.20 ± 3.33	0.433
Parenteral drug use	16 (4.8)	10 (7.9)	6 (2.9)	0.062
*N* of parenteral applications daily (range)	0.12 ± 0.59 (0–4)	0.20 ± 0.76	0.07 ± 0.44	0.068
*N* of comorbidities, mean (range)	3.70 ± 1.76 (1–14)	3.72 ± 1.78	3.68 ± 1.75	0.829
Patients with multimorbidity (without SA/VA)	313 (94.6)	123 (96.9)	190 (93.1)	0.213

AF, atrial fibrillation; LVEF, left ventricular ejection fraction; ACS, acute coronary syndrome; MI, myocardial infarction; CAD, coronary artery disease; PCI, percutaneous coronary intervention; CABG, coronary artery bypass grafting; AFL, atrial flutter; LVEF, left ventricular ejection fraction; TIA, transient ischemic attack; ICD, implantable cardioverter defibrillator; CRT, cardiac resynchronization therapy; COPD, chronic obstructive pulmonary disease; OAC, oral anticoagulant therapy; VKA, vitamin K antagonist; NOAC, non-vitamin K antagonist oral anticoagulant; ASA, acetylsalicylic acid; DHP, dihydropyridine; ACEI, angiotensin converting enzyme inhibitor; ARB, angiotensin receptor inhibitor; PPI, proton pump inhibitor; ECV, electrical cardioversion; SA, supraventricular arrhythmias; VA, ventricular arrhythmias; N, number.

^1^There were no immobile patients in this cohort.

^2^CIED: cardiac implantable electronic devices (antybradicardia PM: n = 20, ICD: n = 7, CRT: n = 5).

^3^Thyroid disfunction: hypothyroidism, n = 65, hyperthyroidism, n = 29.

^4^Anthyarrhythmics: mexiletine, propafenone, flecanide, sotalol, amiodaron.

The analyses of the EQ-5D questionnaire and QoL were conducted in the same manner as the analyses of TB. In addition, the relationship of the EQ-5D score with TB quartiles was analyzed using the Kruskal–Wallis H test.

The statistical software program IBM SPSS Statistics for Windows, version 26 (IBM Corp., Armonk, NY, USA) was used for all analyses. All reported *P*-values in this study were two-sided, and the *P*-value of <0.05 was considered statistically significant.

## Results

### Study population

Of 331 patients with AF, 127 (38.4%) were females, and 204 (61.6%) were males. Sex differences in socio-demographic characteristics, concomitant comorbidities, and concomitant therapy are shown in Table 1.

The mean age was 65.4 ± 10.3 years, the mean CHA_2_DS_2_-VASc score was 2.63 ± 1.50, and the mean total history of AF was 6.41 ± 6.62 years (median 4.00 years, IQR 7.00 years). Permanent AF, multimorbidity, and polypharmacy were reported in 97 (29.3%), 313 (94.6%), and 237 (71.6%) patients, respectively.

Compared with males, females were more likely to be older and more frequently had valvular heart disease or ≥1 non-sex-related CHA_2_DS_2_-VASc score stroke risk factor (all *P* ≤ 0.05). Males were more likely to have left ventricular ejection fraction <50% and ischemic heart disease and more frequently underwent catheter ablation and/or electrical cardioversion (ECV) of AF compared with females (all *P* ≤ 0.037). There were no differences in concomitant therapy, a number of comorbidities, the prevalence of multimorbidity, and polypharmacy between the sexes (see Table 1).

### Sex differences in self-reported treatment burden

**FIGURE 2:**
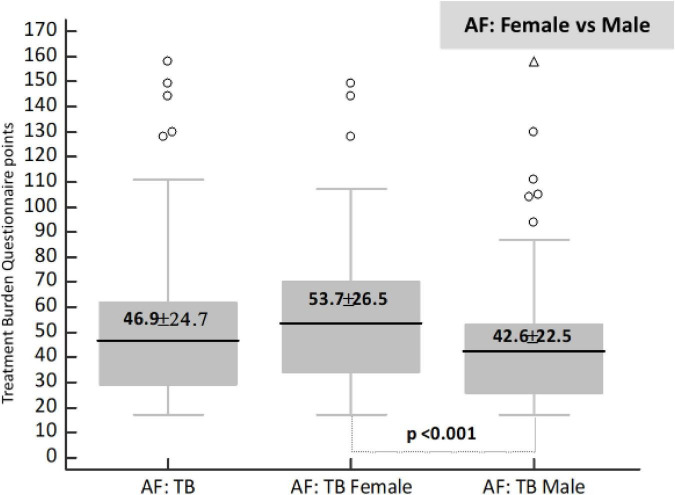
Sex difference in treatment burden in whole and atrial fibrillation (AF) study cohort. TB, treatment burden; AF, atrial fibrillation.

The mean self-reported TB score was 46.9 points (27.6% of the maximum score value of 170 points). The mean TB score was significantly higher in females compared with males [53.7 (31.6% of 170 points) vs. 42.6 (25.1% of 170 points) points, *p* < 0.001]; also, females reported significantly more frequently TB ≥ 59 points compared with males (37.8% vs. 20.6%, *P* = 0.001), see Figure 2 and Table 2.

**TABLE 2:** The distribution of study cohort per quartiles of treatment burden in atrial fibrillation (AF).

Treatment burden	AF patients *N* (%)	Female *N* (%)	Male *N* (%)	*P*-value
Mean value	46.87	53.73	42.59	<0.001
95% CI	44.20–49.53	49.08–58.39	39.49–45.69	
Range	17–158	17–149	17–158	
SD	24.66	26.52	22.45	
Median	40.00	48.00	37.00	
IQR	33.00	36.00	27.00	
Proportion of the maximum 170 points	27.57%	31.61%	25.05%	
**TB quartiles**				
TB ≤ 26 points	73 (22.1)	21 (16.5)	52 (25.5)	0.058
TB 27–39 points	89 (26.9)	27 (21.3)	62 (30.4)	0.070
TB 40–58 points	79 (23.9)	31 (24.4)	48 (23.5)	0.855
TB ≥ 59 points	90 (27.2)	48 (37.8)	42 (20.6)	0.001
Total	331	127 (38.4)	204 (61.6)	

AF, atrial fibrillation; N, number; CI, confidence interval; SD, standard deviation; IQR, interquartile range: TB, treatment burden.

#### Item-specific sex differences in self-reported treatment burden

Both females and males attributed the highest TB to administrative issues, including visit appointments and health-related paperwork, and diet modification requirements, see Supplementary Table 1.

**FIGURE 3:**
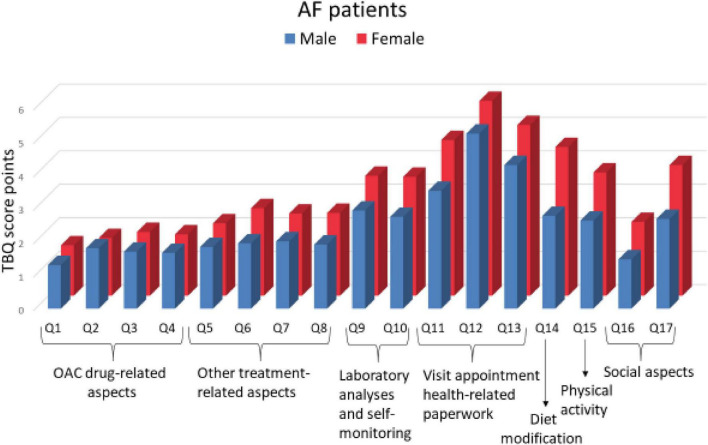
Sex difference in specific management aspect-related treatment burden. AF, atrial fibrillation; TBQ, treatment burden questionnaire; Q, question; OAC, oral anticoagulant.

In comparison to males, females reported significantly higher TB score values for questions about the frequency of drugs intake per day and specific conditions when drugs are taken, self-monitoring, doctor visits, diet modifications, physical activity requirements, and social aspects of TB score (all *P* ≤ 0.05); see Figure 3 and Supplementary Table 1.

#### Sex-specific univariate and multivariable analysis of self-reported treatment burden

The univariate and multivariable analyses of self-reported TB are shown in Table 3 and Supplementary Tables 3–5.

**TABLE 3:** Multivariable linear regression and logistic regression analyses of treatment burden in atrial fibrillation (AF) patients.

	Variable	Beta	95% CI	*P*-value
**Multivariable linear regression analysis**				
Female	VKA therapy	0.192	1.19–19.24	0.027
	Diuretic therapy	–0.219	–21.05–(−2.67)	0.012
Male	Ablation and/or ECV	–0.179	–14.13–(–1.89)	0.011
**Multivariable logistic regression analysis of the highest TB quartile (TB ≥ 59)**				
Female	PPI therapy	5.354	1.97–14.56	0.001
	NOAC	0.319	0.12–0.83	0.019
	Diuretic therapy	0.318	0.13–0.76	0.010
	CHA_2_DS_2_-VASc score	0.700	0.49–0.99	0.045
Male	Ablation and/or ECV	0.383	0.18–0.81	0.012
	Supraventricular arrhythmias	0.222	0.05–0.98	0.047
**Multivariable logistic regression analysis of the lowest TB quartile (TB ≤ 26)**				
Female	PCI/balloon angioplasty	7.642	1.11–52.59	0.039
	Supraventricular arrhythmias	4.155	1.21–14.30	0.024
	Former smoker	3.752	1.15–12.21	0.028
Male	Ablation AF	2.753	1.26–6.01	0.011
	Age ≤ 50 years	0.187	0.04–0.85	0.030

VKA, vitamin K antagonist; ECV, electrical cardioversion; AF, atrial fibrillation; PPI, proton pump inhibitor; PCI, percutaneous coronary intervention; NOAC, non-vitamin K antagonist oral anticoagulant.

Oral anticoagulant treatment duration ranged from 0.5 to 21 years, there were no statistically significant differences in the mean duration of OAC treatment between sexes. On univariate analysis, OAC treatment duration as well as OAC treatment duration less than 1 year were not significantly associated with total TBQ score value, or the highest and the lowest TBQ score quartile in either sex, see Supplementary Tables 3–5. On multivariable linear regression analysis, VKA therapy was statistically significantly associated with higher TB (*p* = 0.001) in females, while diuretic therapy was a negative predictor of higher TB (*p* = 0.012). In males, ablation and/or ECV were negative predictors of higher TB, see Table 3.

On multivariable analysis of the highest TB quartile (TB ≥ 59), NOAC use [Odds Ratio (OR) 0.319; 95% Confidence Interval (CI) 0.12–0.83, *P* = 0.019], diuretic therapy (OR 0.318; 95% CI 0.13–0.76, *P* = 0.010), and CHA_2_DS_2_-VASc score (OR 0.700; 95% CI 0.49–0.99, *P* = 0.045) were negatively associated with the highest TB quartile, while proton pump inhibitor (OR 5.354; 95% CI 1.97–14.56, *P* = 0.001) was positively associated with the highest TB quartile, in females. In males, on a multivariable analysis of the highest TB quartile, catheter ablation and/or ECV of AF (OR 0.383; 95% CI 0.18–0.81, *P* = 0.012), and supraventricular arrhythmias (OR 0.222; 95% CI 0.05–0.98, *P* = 0047) were negatively associated with the highest TB quartile, see Table 3.

On multivariable analyses of the lowest TB quartile, there was a positive association with catheter ablation of AF (OR 2.753; 95% CI 1.26–6.01, *P* = 0.011) and a negative association with age ≤50 (OR 0.187; 95% CI 0.04–0.85, *P* = 0.030) in males, and a positive association with PCI/balloon angioplasty (OR 7.642; 95% CI 1.11–52.59, *P* = 0.039) in females, see Table 3.

On a multivariable sensitivity analysis restricted to patients taking a VKA [*n* = 189 patients, (57.1%)], the female sex was significantly associated with a higher TB score (Beta 0.187; 95% CI 2.4–18.5, *P* = 0.011), and the association was also present in the analysis restricted to 110 patients taking a NOAC (Beta 0.226; 95% CI 2.3–16.0, *P* = 0.009).

On a univariate sensitivity analysis restricted to AF patients without OAC therapy [*n* = 32 patients (9.7%)], there were no significant differences in self-reported TB between the sexes (Beta 0.201; 95% CI -7.3–25.2, *P* = 0.269).

### Sex-related differences in self-reported quality of life and relations with treatment burden

The lowest QoL reflects the highest EQ-5D score of 20 points, whereas the highest QoL would reflect the lowest EQ-5D score of 0 points.

Overall, the mean EQ-5D score value was 2.95 points (14.75% of the maximum 20 points). Females reported significantly higher EQ-5D score (i.e., lower QoL) compared with males (3.97 vs. 2.32 points, *P* < 0.001), Table 4. The EQ-5D item-specific sex differences are shown in Supplementary Table 2. Compared with males, female patients reported significantly higher EQ-5D score for mobility, pain/discomfort, and anxiety/depression (all *P* ≤ 0.002); see Supplementary Table 2.

**TABLE 4:** The distribution of study cohort per quartiles of EQ-5D questionnaire in atrial fibrillation (AF).

	AF patients *N* (%)	Female *N* (%)	Male *N* (%)	*P*-value
**EQ-5D questionnaire**				
Mean value	2.95	3.97	2.32	<0.001
95% CI	2.60–3.31	3.39–4.54	1.90–2.75	
Range (minimum-maximum)	0–15	0–14	0–15	
SD	3.25	3.28	3.08	
Median	2.00	3.00	1.00	
IQR	5.00	5.00	3.00	
Proportion of the maximum 20 points	14.75%	19.85%	11.60%	
**EQ-5D quartiles**				
EQ-5D ≤ 1 point	150 (45.3)	36 (28.3)	114 (55.9)	0.094
EQ-5D = 2 points	39 (11.8)	17 (13.4)	22 (10.8)	<0.001
EQ-5D 3–5 points	83 (25.1)	38 (29.9)	45 (22.1)	<0.001
EQ-5D ≥ 6 points	59 (17.8)	36 (28.3)	23 (11.3)	<0.001
Total	331	127 (38.4)	204 (61.6)	

AF, atrial fibrillation; N, number; CI, confidence interval; SD, standard deviation; IQR, interquartile range.

**FIGURE 4:**
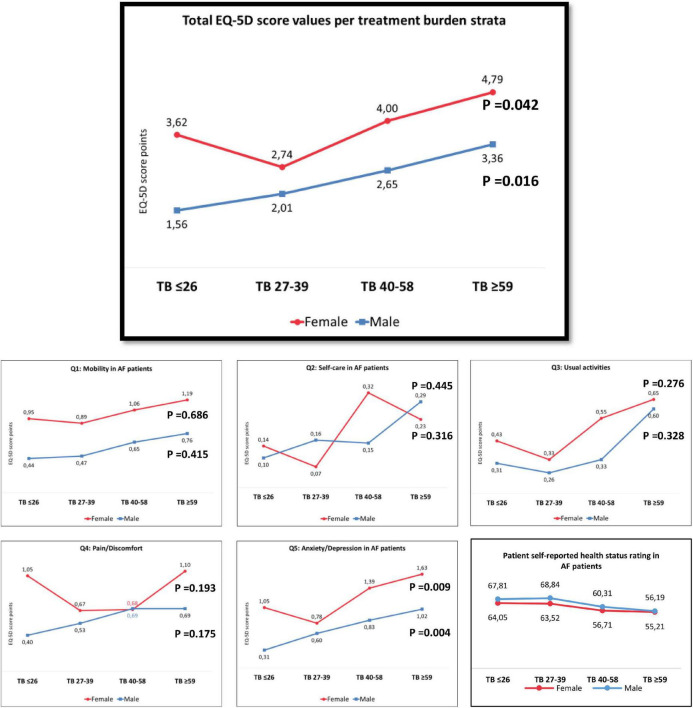
Sex difference in self-reported health rating and the EQ-5D score values across treatment burden quartiles.

With increasing the TB mean score, the mean EQ-5D QOL score significantly increases (i.e., QoL was lower) in both sexes. In addition, the anxiety/depression question score was significantly increased with increasing TB among both sexes (see, Figure 4).

Univariate and multivariable analyses of the EQ-5D score are shown in Supplementary Tables 6–9. On multivariable analyses, the highest TB quartile (Beta 0.182; 95% CI 0.25–2.20, *P* = 0.015), question about physical activity requirements within TB (Beta 0.237, 95% CI 0.13–0.41, *P* < 0.001), age (Beta 0.156; 95% CI 0.01–0.11, *p* = 0.032), and mobility with help (Beta 0.149; 95% CI 0.06–3.73, *P* = 0.043) were associated with the lower QoL score in females, whereas number of comorbidities (Beta 0.160; 95% CI 0.09–0.473, *P* = 0.004), peripheral arterial disease (Beta 0.297; 95% CI 6.04–12.53, *P* < 0.001), physical activity requirements within TB (Beta 0.297; 95% CI 0.22–0.45, *P* < 0.001), and the social aspect of TB (Beta 0.186; 95% CI 0.16–0.56, *P* = 0.001) were associated with the lower QoL score in males, see Supplementary Table 10.

#### Overall self-rated health status

Females reported significantly lower self-estimated health status ratings than males (58.8 ± 19.72 vs. 64.0 ± 20.6, *p* = 0.025).

The self-estimated status rating decreased with increasing TB score in the entire study cohort [Beta 0.200; 95% CI -0.25–(-0.08), *P* < 0.001] and among males [Beta -0.224; 95% CI -0.33–(-0.08), *P* = 0.001], but not when the analysis was restricted to females (Beta -0.116; 95% CI -0.22–0.05, *P* = 0.195).

The highest TB quartile (≥59 points) was significantly associated with the lowest self-reported health status rating quartile in the whole cohort (OR 2.185; 95% CI 1.33–3.58, *P* = 0.002) and among females (OR 2.100; 95% CI 1.01–4.35, *P* = 0.046), but the association was of borderline significance on the analysis restricted to males (OR 1.962; 95% CI 0.98–3.93, *P* = 0.057); see Supplementary Table 10.

## Discussion

To our knowledge, this study was the first to compare the TB, explore its significant determinants, and a TB impact on QoL between sexes in AF patients. The main findings were as follows: (i) females reported significantly higher TB compared to males; (ii) approximately 1 in 5 males and 2 in 5 females reported TB ≥ 59 points.

In detail, our analysis showed that: the most considerable share of self-reported TB in both sexes was attributed to administrative issues (e.g., visit appointments, health-related paperwork) and diet modification requirements; Compared with males, females reported significantly higher TB for questions about frequency of drugs intake per day and specific conditions drugs are taken, self-monitoring, doctor visits, diet modifications, physical activity requirements, and social aspects of TB score; Females who were taking VKA reported significantly higher TB than those without, while NOAC use in females was a negative predictor of the TB ≥ 59 points, such finding were not recorded in males; In males ablation of AF and in females PCI and/or balloon angioplasty were positive predictors of TB ≤ 26 points; The highest TB quartile (≥59 points) and TB question about physical activity were significantly associated with lower QoL in females, while TB questions about physical activity and social aspects were significantly associated with lower QoL in males; Questions about a doctor visit appointment and recommended physical activity were associated with the highest EQ-5D quartile (score ≥ 6 points) in females, while question-related to recommended physical activity was significantly associated with the highest EQ-5D quartile; The highest TB quartile was significantly associated with the lowest self-reported health status rating quartile in females, but the association was of borderline significance when the analysis was restricted to males.

Our analysis showed that females were significantly older than males, which aligns with previously published studies (21–23). Similarly, to other reports, females in our study underwent significantly less invasive procedures than males, also shown in several other studies (23–25).

A recent study of non-AF patients with various chronic health conditions reported that a TB of ≥59 points was unacceptably high for patients (14). In our main study, we determined that the highest quartile of TBQ among patients with AF was also ≥59 points and reported that 1 in 4 patients with AF has TB ≥ 59 points (15). In the current analysis, we showed that 2 in 5 females with AF reported a TB ≥ 59 points compared to 1 in 5 males, suggesting that females are more burdened by AF treatment than males. Furthermore, multivariable analysis restricted to non-AF patients showed no significant difference in TB among sexes, suggesting that the AF management burdened significantly more females than males. Similarly, the recent study which explored treatment burden among multimorbid patients using the Multimorbidity TBQ also showed a higher TB score among females (26).

The higher TB score in females may be influenced by a higher severity of symptoms, higher heart rate in AF, and longer durations of AF episodes, which occur more frequently in females compared to males, and thus requiring more frequent healthcare visits (23, 27). Females also underwent catheter ablation significantly less often and remained on antiarrhythmic drug therapy longer than males, which could also contribute to the higher TB (23).

Our main study findings suggest that it is essential to improve the healthcare system organization, as it may diminish TB in AF patients (15). Current analysis suggests that females may be more affected by TB than males, therefore, females may benefit more from better system organization.

Our findings also suggest that OAC use is more burdensome for females compared with males, and using a NOAC rather than a VKA in females could decrease TB below the unacceptable TB. Regarding males, a rhythm control strategy for AF management could significantly decrease TB.

Females reported significantly higher TB score on specific questions regarding self-monitoring, doctor visits, diet modifications, physical activity, and social aspects of TB. These particular areas may be the primary goal of developing sex-specific interventions and strategies to improve the healthcare system to reduce TB in females.

It has been previously reported that impaired QoL was associated with increased morbidity and mortality among patients with chronic cardiac health conditions (28). The recently published systematic review of sex differences in QoL in AF patients suggests that lower QoL in females may be explained by a more substantial effect of AF on females than on males (29). In line with other studies, we showed that females with AF have lower QoL than males with AF (30). In our main study, female sex, and TB were independent predictors of lower QoL in AF patients but not in non-AF patients, suggesting that AF may be more burdensome to females than males (15). Current analysis provides new insights regarding the impact of TB on quality of life. In females, TB ≥ 59 and TB question regarding recommended physical activity, while in males, recommended physical activity and social aspects of TB were independent drivers of the lower QOL. Thus, improving the burden of treatment or using a minimally disruptive medicine approach could lower TB and improve QoL, especially among females, but that needs further investigation.

In our study, females reported significantly lower self-reported health status than males. These sex differences in self-reported health status were also reported in an extensive global survey conducted in 59 countries and were attributed to possibly combined biological and social factors (31). We found that TB scores ≥59 points were an independent predictor of lower self-reported health status in AF patients, also when the analysis was restricted to females but not in males, implying that lowering TB using a minimally disruptive medicine approach may also lead to an improvement in self-reported health status.

## Limitations

Single-center study results may not be generalizable to other AF cohorts. Nevertheless, the BALKAN-AF study, including Serbia, showed that the cardiovascular and AF-related risk profile of AF patients was broadly similar to AF patients in other European countries (32). We prospectively included consecutive patients with AF, but the relatively small cohort size may have influenced the results. In addition, in our study, we did not collect data on other factors that could influence TB, such as, for example, patient knowledge of AF, mental status, cognitive function, etc. Owing to the use of modified TBQ in this study, our findings may not be comparable to studies using originally reported TBQ. Nevertheless, the aim of our study was not a comparison to other chronic medical conditions, but the analysis of sex-related differences in self-reported TB among patients with AF. Also, our study did not investigate the financial burden of treatment because of the nationwide health insurance system used by all citizens in Serbia. Another limitation of our study is the lack of follow-up, as the treatment burden may change over time, but the follow-up in our study is ongoing.

## Conclusion

Our study was the first to explore sex-specific determinants of TB in AF patients. Females reported significantly higher TB compared with males. A TB of ≥59 points (i.e., unacceptably high TB) was reported by 2 out of 5 females and 1 out of 5 males with AF. Using a NOAC rather than VKA in females and a rhythm control strategy in males could decrease TB to acceptable values. More research is needed to confirm our findings in different AF cohorts and elucidate how to decrease TB in patients with AF.

## Data availability statement

The original contributions presented in this study are included in the article/Supplementary material, further inquiries can be directed to the corresponding author.

## Ethics statement

The studies involving human participants were reviewed and approved by the School of Medicine, Belgrade University, Ethical Committee. The patients/participants provided their written informed consent to participate in this study.

## Author contributions

TP: study design, manuscript preparation, and critical intellectual impact. MMi: data acquirement and drafting of the manuscript. JS, MMa, VK, and AK: data acquirement and manuscript reviewing. NM: manuscript reviewing. All authors contributed to the article and approved the submitted version.
