# Supplementary material for: Sex-related differences in self-reported treatment burden in patients with atrial fibrillation
Source: Front Cardiovasc Med. 2022 Nov 4;9:1029730. doi: 10.3389/fcvm.2022.1029730 (PMC9671945; doi:10.3389/fcvm.2022.1029730)
Supplement: Supplementary file 1 [file Table_1.DOCX]

**Appendix.**

**Sex-related differences in self-reported treatment burden in patients with atrial fibrillation**

Miroslav Mihajlovic^1,2^, Jelena Simic^1^, Milan Marinkovic^2^, Vladan Kovacevic^2^, Aleksandar Kocijancic^2^, Nebojsa Mujovic^1,2^, Tatjana S. Potpara^1,2^

1 School of Medicine, Belgrade University, Belgrade, Serbia.

2 Cardiology Clinic, University Clinical Centre of Serbia, Belgrade, Serbia.

**Address for correspondence**

Associate Professor Tatjana S. Potpara, MD, PhD

School of Medicine, Belgrade University

Dr Subotica 8, 11 000 Belgrade,

Serbia Tel/Fax: +381 11 3616319

Email address: tatjana.potpara@med.bg.ac.rs

**Table S1. Sex differences in treatment burden questionnaire (TBQ) score values in AF and non-AF patients.**

| **Questions** | **AF**  n=331 (%) | **Female**  n=127 (38.4) | **Male**  n=204 (61.6) | **P value** |
| --- | --- | --- | --- | --- |
| ***Questions about OAC-related treatment burden*** |  |  |  |  |
| 1. The taste, shape or size of your tablets and/or the inconvenience caused by your injections (for example, pain, bleeding, scars) | 1.38 ±1.30 | 1.51 ±1.55 | 1.31 ±1.12 | 0.158 |
| 2. The number of times you have to take your medication every day | 1.78 ±2.00 | 1.73 ±1.92 | 1.81 ±2.05 | 0.719 |
| 3. The things you do to remind yourself to take your daily medication and/or to manage your treatment when you are not at home | 1.79 ±1.98 | 1.91 ±2.06 | 1.71 ±1.93 | 0.386 |
| 4. The specific conditions when taking your medication (for example, taking it at a specific time of the day or meal, not being able to do certain things after taking them like driving or lying down) | 1.73 ±1.91 | 1.84 ±2.07 | 1.67 ±1.81 | 0.430 |
| ***Questions about other drugs-related treatment burden ^a^*** |  |  |  |  |
| 1. The taste, shape or size of your tablets and/or the inconvenience caused by your injections (for example, pain, bleeding, scars) | 1.98 ±2.15 | 2.17 ±2.35 | 1.85 ±2.00 | 0.187 |
| 2. The number of times you have to take your medication every day | 2.21 ±2.37 | 2.62 ±.75 | 1.96 ±2.07 | 0.013 |
| 3. The things you do to remind yourself to take your daily medication and/or to manage your treatment when you are not at home | 2.19 ±2.38 | 2.46 ±2.60 | 2.02 ±2.23 | 0.099 |
| 4. The specific conditions when taking your medication (for example, taking it at a specific time of the day or meal, not being able to do certain things after taking them like driving or lying down) | 2.13 ±3.04 | 2.48 ±2.74 | 1.92 ±2.14 | 0.037 |
| ***Questions about other aspects of treatment burden*** |  |  |  |  |
| 1. Lab tests and other exams (frequency, time spent and inconvenience of these exams) | 3.19 ±3.04 | 3.59 ±3.30 | 2.94 ±2.85 | 0.059 |
| 2. Self-monitoring (for example, INR controls, taking your blood pressure or measuring your blood sugar yourself: frequency, time spent and inconvenience of this surveillance) | 3.06 ±3.01 | 3.56 ±3.39 | 2.75 ±2.71 | 0.017 |
| 3. Doctor visits (frequency and time spent for the visits) | 3.97 ±3.36 | 4.66 ±3.46 | 3.53 ±3.22 | 0.003 |
| 4. Arrange appointments and schedule doctor visits and laboratory tests | 5.46 ±3.86 | 5.83 ±3.84 | 5.24 ±3.86 | 0.175 |
| 5. How would you rate the burden associated with taking care of paperwork from health insurance agencies, welfare organizations, hospitals and/or social care? | 4.61 ±3.77 | 5.11 ±3.96 | 4.30 ±3.62 | 0.175 |
| 6. How would you rate the constraints associated with your diet (for example, not being allowed to eat certain foods)? | 3.42 ±3.16 | 4.45 ±3.45 | 2.78 ±2.78 | <0.001 |
| 7. How would you rate the burden associated with the recommendations from your doctors to practice regular physical exercises? | 3.04 ±3.04 | 3.69 ±3.34 | 2.64 ±2.77 | 0.002 |
| 8. What is the impact of your healthcare on your social relationships (for example, need for assistance, being ashamed to take your medication in front of people)? | 1.76 ±2.07 | 2.21 ±2.61 | 1.48 ±1.59 | 0.002 |
| 9. 'Frequent healthcare reminds me of my health problems' | 3.16 ±3.06 | 3.91 ±3.43 | 2.69 ±2.72 | <0.001 |

**OAC**: Oral anticoagulant therapy; **INR:** International normalized ratio.

^a^ This section represent modified TBQ questions.

Table S2. Sex differences in Qualitive of life 5D questionare score values in AF and non-AF patients.

| **Questions** | **AF**  n=331 (%) | **Female**  n=127 (38.4) | **Male**  n=204 (61.6) | **P value** |
| --- | --- | --- | --- | --- |
| Q 1 –Mobility | 0.75 ±1.06 | 1.06 ±1.10 | 0.56 ±0.99 | <0.001 |
| Q 2 -Self-care | 0.18 ±0.63 | 0.20 ±0.63 | 0.17 ±0.62 | 0.591 |
| Q 3 -Usual activities | 0.42 ±0.92 | 0.52 ±0.98 | 0.36 ±0.89 | 0.121 |
| Q 4 -Pain/Discomfort | 0.69 ±0.95 | 0.90 ±1.01 | 0.57 ±0.89 | 0.002 |
| Q 5 -Anxiety/Depression | 0.91 ±1.06 | 1.29 ±1.11 | 0.67 ±0.95 | <0.001 |
| QoL EQ-5D total score | 2.95 ±3.25 | 3.97 ±3.28 | 2.32 ±3.08 | <0.001 |
| Overall self-rated health status today  (Range: 0-100) | 61.98 ±20.39 | 58.80 ±19.72 | 63.97 ±20.61 | 0.025 |

**AF:** Atrial fibrillation; **Q:** Question.

**Table S3. Univariate analyses of treatment burden as a continuous variable in patients with atrial fibrillation.**

| **Variable** | **Patients with AF**  **n=331 (%)** | | | **Female**  **n=127 (38.4)** | | | **Male**  **n=204 (61.6)** | | |
| --- | --- | --- | --- | --- | --- | --- | --- | --- | --- |
|  | **beta** | **CI 95%** | **P value** | **beta** | **CI 95%** | **P value** | **beta** | **CI 95%** | **P value** |
| Age | -0.059 | -0.40-0.12 | 0.286 | -0.172 | -1.05-0.01 | 0.053 | -0.069 | -0.43-0.14 | 0.325 |
| Age ≤40 | 0.002 | -18.13-19.00 | 0.964 | 0.068 | -32.35-73.21 | 0.445 | 0.002 | -18.14-18.64 | 0.979 |
| Age ≤45 | 0.024 | -10.04-15.63 | 0.669 | 0.037 | -24.35-37.19 | 0.680 | 0.048 | -8.60-17.78 | 0.493 |
| Age ≤47 | 0.066 | -4.45-18.46 | 0.230 | 0.048 | -19.50-34.00 | 0.593 | 0.112 | -2.18-21.48 | 0.109 |
| Age ≤48 | 0.040 | -6.51-14.07 | 0.470 | 0.048 | -19.50-34.00 | 0.593 | 0.084 | -4.09-16.74 | 0.232 |
| Age ≤49 | 0.027 | -7.44-12.40 | 0.623 | 0.048 | -19.50-34.00 | 0.593 | 0.069 | -5.00-14.98 | 0.326 |
| Age ≤50 | 0.023 | -7.32-11.28 | 0.676 | 0.014 | -22.10-25.00 | 0.873 | 0.078 | -4.11-14.79 | 0.266 |
| Age ≤64 | 0.061 | -2.36-8.51 | 0.267 | 0.178 | 0.20-20.62 | 0.046 | 0.063 | -3.39-9.03 | 0.372 |
| Age 65-74 | -0.035 | -7.13-3.66 | 0.528 | 0.005 | -9.10-9.60 | 0.958 | -0.111 | -11.49-1.22 | 0.113 |
| Age ≥75 | -0.034 | -9.28-4.86 | 0.538 | -0.199 | -23.72-(-1.61) | 0.025 | 0.065 | -4.71-13.05 | 0.355 |
| Age ≥80 | 0.017 | -8.53-11.68 | 0.760 | -0.047 | -23.06-13.35 | 0.599 | 0.068 | -5.87-17.20 | 0.334 |
| *Education degree* | | | | | | | | | |
| Elementary | -0.086 | -14.11-1.57 | 0.117 | -0.102 | -18.80-4.97 | 0.252 | -0.135 | -20.55-0.16 | 0.054 |
| High school | 0.070 | -1.90-8.76 | 0.206 | 0.086 | -4.79-13.85 | 0.338 | 0.063 | -3.39-9.02 | 0.372 |
| College | 0.107 | -0.09-15.13 | 0.053 | 0.146 | -2.21-24.32 | 0.101 | 0.083 | -3.53-14.21 | 0.237 |
| University | -0.102 | -12.35-0.35 | 0.064 | -0.142 | -22.13-2.33 | 0.112 | -0.046 | -9.43-4.72 | 0.512 |
| *Employment status* | | | | | | | | | |
| Employed | -0.048 | -8.90-3.40 | 0.380 | 0.066 | -9.30-20.50 | 0.458 | -0.019 | -7.49-5.65 | 0.783 |
| Unemployed | 0.006 | -9.89-11.11 | 0.909 | -0.066 | -23.76-10.89 | 0.463 | 0.053 | -7.83-17.57 | 0.450 |
| Retired | 0.042 | -3.53-7.91 | 0.451 | -0.008 | -12.48-11.41 | 0.929 | -0.008 | -6.69-5.98 | 0.912 |
| *Marital status* | | | | | | | | | |
| Married/living with a partner | -0.097 | -11.74-0.63 | 0.078 | -0.101 | -15.55-4.22 | 0.259 | -0.033 | -9.76-6.03 | 0.642 |
| Alone/divorced | 0.055 | -4.45-13.60 | 0.319 | 0.131 | -4.62-31.51 | 0.143 | 0.039 | -7.06-12.58 | 0.580 |
| Widow(er) | 0.072 | -2.53-12.47 | 0.193 | 0.032 | -8.74-12.57 | 0.722 | 0.002 | -11.39-11.73 | 0.977 |
| *Cigarette smoking* | | | | | | | | | |
| Smoker  Former smoker  Non-smoker | -0.002 | -7.65-7.39 | 0.973 | 0.012 | -13.91-15.95 | 0.892 | 0.020 | -7.03-9.46 | 0.771 |
|  | 0.028 | -4.39-7.48 | 0.609 | 0.025 | -10.84-14.33 | 0.784 | 0.108 | -1.39-11.54 | 0.123 |
|  | -0.024 | -6.60-4.19 | 0.660 | -0.029 | -12.17-8.75 | 0.747 | -0.119 | -11.52-0.84 | 0.090 |
| *Functional mobility* | | | | | | | | | |
| Fully mobile  Mobile with help  Immobile | -0.088 | -21.33-2.13 | 0.108 | -0.081 | -26.46-9.86 | 0.368 | -0.075 | -23.30-6.88 | 0.284 |
|  | 0.088 | -2.13-21.33 | 0.108 | 0.081 | -9.86-26.46 | 0.368 | 0.075 | -6.88-23.20 | 0.284 |
|  | ----- | ----- | ----- | ----- | ----- | ----- | ----- | ----- | ----- |
| *AF characteristics* | | | | | | | | | |
| Total AF history (years) | 0.042 | -0.25-0.56 | 0.454 | 0.049 | -0.63-1.10 | 0.591 | 0.077 | -0.19-0.68 | 0.276 |
| Permanent AF | 0.002 | -5.78-5.95 | 0.977 | -0.083 | -15.06-5.45 | 0.355 | 0.065 | -3.63-9.98 | 0.359 |
| *Comorbid conditions* | | | | | | | | | |
| Hypertension | -0.011 | -7.64-6.22 | 0.840 | -0.126 | -22.87-3.73 | 0.157 | 0.031 | -5.98-9.39 | 0.662 |
| Heart failure | -0.066 | -14.17-3.39 | 0.228 | 0.002 | -18.05-18.39 | 0.985 | -0.081 | -14.95-3.94 | 0.252 |
| LVEF <50% | -0.131 | -16.40-(-1.62) | 0.017 | -0.090 | -33.21-10.69 | 0.312 | -0.091 | -12.48-2.57 | 0.195 |
| *Ischemic heart disease* | -0.117 | -14.55-(-0.597) | 0.033 | -0.183 | -29.21-(-0.72) | 0.040 | -0.045 | -10.07-5.15 | 0.525 |
| Recent ACS | 0.038 | -22.23-46.64 | 0.486 | 0.116 | -18.01-87.09 | 0.196 | -0.039 | -57.11-31.80 | 0.575 |
| Prior MI | -0.093 | -17.46-1.35 | 0.093 | -0.140 | -54.79-6.18 | 0.117 | -0.034 | -11.58-7.05 | 0.632 |
| Chronic stable CAD | -0.073 | -20.82-4.02 | 0.184 | -0.017 | -22.47-18.50 | 0.848 | -0.133 | -29.45-0.55 | 0.059 |
| PCI/Balloon angioplasty | -0.067 | -15.00-3.56 | 0.226 | -0.178 | -43.80-(-0.42) | 0.046 | 0.022 | -8.14-11.14 | 0.759 |
| CABG | -0.045 | -24.54-10.21 | 0.418 | ----- | ----- | ----- | -0.025 | -18.83-13.17 | 0.728 |
| Cardiomyopathy | -0.038 | -12.52-6.07 | 0.495 | 0.027 | -17.33-23.64 | 0.761 | -0.041 | -12.69-6.94 | 0.564 |
| Valvular disease | 0.021 | 8.14-12.07 | 0.703 | 0.007 | -13.57-14.61 | 0.942 | -0.036 | -19.00-11.25 | 0.614 |
| Supraventricular arrhythmias (SA+AFL) | -0.145 | -17.10-(-2.58) | 0.008 | -0.159 | -25.29-1.18 | 0.074 | -0.131 | -16.14-0.39 | 0.062 |
| Ventricular arrhythmias | -0.041 | -12.81-5.77 | 0.457 | 0.055 | -12.53-23.86 | 0.539 | -0.083 | -16.32-4.06 | 0.237 |
| *CIEDs* | 0.063 | -4.19-15.98 | 0.251 | 0.110 | -6.82-29.40 | 0.220 | 0.039 | -8.26-14.84 | 0.575 |
| Anti-bradycardia pacemaker | 0.090 | -1.19-20.96 | 0.102 | 0.104 | -8.30-32.45 | 0.243 | 0.087 | -4.84-21.47 | 0.214 |
| ICD | -0.038 | -46.37-22.00 | 0.496 | -0.002 | -53.64-52.17 | 0.978 | -0.080 | -70.07-18.63 | 0.254 |
| CRT | -0.012 | -27.09-21.79 | 0.831 | 0.051 | -37.44-68.22 | 0.565 | -0.036 | -32.49-19.11 | 0.610 |
| Peripheral artery disease | 0.004 | -23.55-25.33 | 0.943 | 0.078 | -20.92-53.97 | 0.384 | -0.076 | -48.71-14.18 | 0.280 |
| Diabetes mellitus type II | 0.051 | -3.59-9.92 | 0.357 | 0.003 | -11.23-11.63 | 0.973 | 0.075 | -3.68-12.40 | 0.286 |
| Prior stroke/TIA | -0.046 | -19.52-7.95 | 0.408 | -0.041 | -25.18-15.77 | 0.650 | -0.082 | -29.24-7.41 | 0.242 |
| Chronic kidney disease | -0.014 | -10.82-8.38 | 0.803 | 0.049 | -13.87-24.58 | 0.582 | -0.030 | -12.71-8.18 | 0.669 |
| COPD | 0.089 | -1.85-18.67 | 0.108 | 0.102 | -7.68-28.58 | 0.256 | 0.086 | -4.51-19.21 | 0.223 |
| Malignancy | 0.080 | -3.18-20.94 | 0.148 | 0.001 | -20.36-20.62 | 0.990 | 0.139 | 0.16-28.67 | 0.047 |
| Thyroid dysfunction | 0.015 | -5.63-7.45 | 0.784 | -0.040 | -13.08-8.23 | 0.653 | 0.023 | -6.71-9.42 | 0.741 |
| Hyperlipoproteinemia | -0.022 | -6.74-4.50 | 0.685 | -0.075 | -14.02-5.69 | 0.404 | 0.020 | -5.60-7.45 | 0.780 |
| Other conditions | 0.043 | -5.04-11.70 | 0.434 | 0.044 | -11.60-19.22 | 0.625 | 0.057 | -5.58-13.34 | 0.419 |
| CHA_2_DS_2_-VASc score | -0.011 | -1.98-1.60 | 0.836 | -0.226 | -8.47-(-1.14) | 0.011 | -0.039 | -2.75-1.54 | 0.579 |
| >1 non-sex CHA_2_DS_2_-VASc risk factors | 0.021 | -7.39-10.94 | 0.703 | -0.102 | -34.63-9.22 | 0.254 | 0.038 | -6.88-12.06 | 0.590 |
| *Current medication* | | | | | | | | | |
| OAC | 0.031 | -6.45-11.62 | 0.574 | 0.055 | -10.23-19.58 | 0.536 | 0.028 | -8.78-13.13 | 0.696 |
| VKA | 0.152 | 2.24-12.91 | 0.006 | 0.210 | 1.94-20.32 | 0.018 | 0.130 | -0.35-12.15 | 0.064 |
| NOAC | -0.141 | -12.96-(-1.73) | 0.010 | -0.184 | -19.96-(-0.54) | 0.039 | -0.120 | -12.28-0.86 | 0.088 |
| OAC treatment duration (years) | 0.093 | -0.14-1.36 | 0.112 | 0.159 | -0.25-2.81 | 0.101 | 0.086 | -0.33-1.29 | 0.242 |
| OAC treatment duration less than 1 year | -0.094 | -11.14-1.1 | 0.108 | -0.138 | -18.7-3.00 | 0.155 | -0.086 | -11.34-2.90 | 0.243 |
| ASA | 0.004 | -8.34-9.02 | 0.939 | -0.052 | -19.27-10.56 | 0.564 | 0.042 | -7.11-13.31 | 0.549 |
| P2Y_12_ inhibitor | 0.011 | -8.72-10.79 | 0.835 | -0.029 | -22.42-16.06 | 0.744 | 0.057 | -6.28-15.07 | 0.418 |
| Beta blocker | -0.092 | -12.23-1.01 | 0.096 | -0.111 | -18.29-4.13 | 0.214 | -0.067 | -11.68-4.08 | 0.343 |
| Non-DHP Ca blocker | 0.080 | -4.52-30.15 | 0.147 | 0.070 | -18.49-42.94 | 0.432 | 0.092 | -6.68-33.34 | 0.190 |
| Digitalis | 0.048 | -7.37-19.13 | 0.383 | -0.003 | 24.39-23.70 | 0.977 | 0.090 | -5.23-24.91 | 0.199 |
| Antiarrhythmic drugs | -0.002 | -5.50-5.35 | 0.978 | 0.032 | -7.88-11.33 | 0.723 | -0.041 | -8.15-4.41 | 0.557 |
| Mexiletine | ---- | ---- | ---- | ---- | ---- | ---- | ---- | ---- | ---- |
| Propafenone | 0.004 | -8.24-8.91 | 0.939 | -0.024 | -16.45-12.52 | 0.789 | 0.017 | -8.97-11.47 | 0.810 |
| Flecainide | -0.001 | -9.81-9.70 | 0.991 | 0.053 | -13.46-24.98 | 0.554 | -0.014 | -11.75-9.63 | 0.845 |
| Sotalol | 0.078 | -7.77-48.41 | 0.150 | 0.061 | -24.52-50.46 | 0.495 | 0.080 | -18.82-69.88 | 0.258 |
| Amiodarone | -0.019 | -6.44-4.51 | 0.726 | 0.006 | -9.20-9.78 | 0.851 | -0.056 | -9.02-3.82 | 0.425 |
| DHP Ca blocker | 0.038 | -3.69-7.61 | 0.496 | -0.083 | -13.86-4.99 | 0.354 | 0.076 | -3.13-10.68 | 0.282 |
| ACEI/ARB | 0.007 | -5.47-6.19 | 0.904 | -0.053 | -13.07-7.03 | 0.553 | 0.063 | -3.74-9.94 | 0.373 |
| Diuretic | -0.108 | -10.72-0.00 | 0.050 | -0.235 | -21.98-(-3.38) | 0.008 | -0.046 | -8.30-4.16 | 0.513 |
| Spironolactone | -0.047 | -8.91-3.50 | 0.392 | 0.067 | -7.27-16.20 | 0.453 | -0.092 | -11.54-2.32 | 0.192 |
| Statins | 0.045 | -3.15-7.70 | 0.410 | -0.003 | -9.55-9.25 | 0.975 | 0.058 | -3.71-9.06 | 0.410 |
| Sedative | 0.082 | -2.08-15.23 | 0.136 | 0.079 | -8.48-22.27 | 0.376 | 0.092 | -3.36-16.60 | 0.192 |
| PPI | 0.091 | -0.92-10.83 | 0.098 | 0.145 | -1.82-19.50 | 0.103 | 0.082 | -2.72-10.69 | 0.242 |
| Insulin | 0.069 | -5.01-22.42 | 0.213 | 0.063 | -12.39-26.03 | 0.483 | 0.034 | -15.16-25.01 | 0.629 |
| Oral antidiabetic drug | 0.043 | -4.51-10.39 | 0.439 | 0.018 | -11.10-13.61 | 0.841 | 0.045 | -6.11-11.94 | 0.525 |
| Other medications | 0.097 | -0.57-10.36 | 0.079 | 0.118 | -3.09-15.65 | 0.187 | 0.054 | -3.98-9.01 | 0.445 |
| *Non-pharmacological treatment* | | | | | | | | | |
| Ablation/ECV | -0.135 | -12.14-(-1.39) | 0.014 | 0.008 | -9.68-10.60 | 0.929 | -0.179 | -14.1-(-1.89) | 0.011 |
| ECV AF | -0.168 | -14.90-(-3.30) | 0.002 | -0.135 | -20.96-2.71 | 0.130 | -0.137 | -12.87-0.01 | 0.050 |
| ECV AFL | -0.020 | -26.02-17.76 | 0.711 | -0.025 | -42.86-32.23 | 0.780 | -0.020 | -29.46-22.16 | 0.781 |
| AF Ablation | -0.045 | -10.15-4.19 | 0.414 | 0.131 | -3.40-23.18 | 0.143 | -0.145 | -16.40-(-0.44) | 0.039 |
| AFL Ablation | -0.125 | -35.23-(-2.65) | 0.023 | ---- | ---- | ---- | -0.136 | -29.79-0.19 | 0.053 |
| Ablation other arrhythmias | 0.008 | -31.81-37.11 | 0.880 | 0.041 | -40.49-65.22 | 0.644 | -0.030 | -54.11-34.83 | 0.670 |
| *Multimorbidity and polypharmacy* | | | | | | | | | |
| Polypharmacy | 0.028 | -4.38-7.46 | 0.610 | -0.043 | -13.49-8.26 | 0.635 | 0.051 | -4.23-9.21 | 0.466 |
| N of drugs | 0.061 | -0.42-1.52 | 0.266 | -0.001 | -1.76-1.73 | 0.987 | 0.088 | -0.40-1.83 | 0.209 |
| N of pills | 0.016 | -0.70-0.94 | 0.771 | -0.033 | -1.82-1.25 | 0.715 | 0.087 | -0.43-1.91 | 0.213 |
| N of drugs without OAC | 0.055 | -0.50-1.54 | 0.314 | -0.014 | -1.97-1.68 | 0.874 | 0.087 | -0.43-1.91 | 0.213 |
| N of pills without OAC | 0.014 | -0.73-0.94 | 0.807 | -0.039 | -1.89-1.21 | 0.665 | 0.033 | -0.72-1.16 | 0.642 |
| Parenteral drug use | 0.042 | -7.64-17.24 | 0.448 | 0.024 | -15.00-19.71 | 0.789 | 0.011 | -16.94-19.84 | 0.877 |
| N of parenteral applications daily | 0.039 | -2.94-6.19 | 0.483 | 0.049 | -4.47-7.88 | 0.586 | -0.029 | -8.59-5.61 | 0.679 |
| N of comorbidities | -0.066 | -2.44-0.59 | 0.232 | -0.128 | -4.53-0.70 | 0.150 | -0.027 | -2.12-1.43 | 0.699 |
| Patients with multimorbidity (without SA/ VA) | 0.021 | -8.86-13.05 | 0.707 | -0.148 | -43.84-3.72 | 0.098 | 0.084 | -4.50-18.54 | 0.231 |

**AF:** Atrial fibrillation; **LVEF:** Left ventricular ejection fraction; **ACS:** Acute coronary syndrome; **MI:** Myocardial infarction; **CAD:** Coronary artery disease; **PCI:** Percutaneous coronary intervention; **CABG:** Coronary artery bypass grafting; **AFL:** Atrial flutter; **CIED:** Cardiac implantable electronic devices; **ICD:** Implantable cardioverter defibrillator; **CRT:** Cardiac resynchronisation therapy; **TIA:** Transient ischemic attack, **COPD:** Chronic obstructive pulmonary disease; **OAC:** Oral anticoagulant therapy; **VKA:** Vitamin K antagonist; **NOAC:** Non-vitamin K antagonist oral anticoagulant; **ASA:** Acetylsalicylic acid; **DHP:** Dihydropyridine; **ACEI:** Angiotensin converting enzyme inhibitor; **ARB:** Angiotensin receptor inhibitor; **PPI:** Proton pump inhibitor; **ECV:** Electrical cardioversion; **SA:** Supraventricular arrhythmias; **VA:** Ventricular arrhythmias; **N:** Number.

**Table S4. Univariate analyses of the highest quartile of the Treatment Burden Questionnaire score in patients with atrial fibrillation.**

| **Variable** | **TBQ ≥59 points**  **Patients with AF**  **N=90 (%)** | | | **TBQ ≥59 points**  **Females**  **n=48 (53.3)** | | | **TBQ ≥59 points**  **Males**  **N=42 (46.7)** | | |
| --- | --- | --- | --- | --- | --- | --- | --- | --- | --- |
|  | **OR** | **CI 95%** | **P value** | **OR** | **CI 95%** | **P value** | **OR** | **CI 95%** | **P value** |
| Age | 0.988 | 0.97-1.01 | 0.299 | 0.947 | 0.91-0.99 | 0.015 | 0.994 | 0.96-1.03 | 0.724 |
| Age ≤40 | 1.073 | 0.20-5.63 | 0.934 | 0.000 | 0.00---- | 1.000 | 0.766 | 0.09-6.74 | 0.810 |
| Age ≤45 | 1.841 | 0.64-5.33 | 0.260 | 3.391 | 0.30-38.44 | 0.324 | 2.026 | 0.58-7.08 | 0.269 |
| Age ≤47 | 2.567 | 1.01-6.54 | 0.048 | 5.200 | 0.53-51.49 | 0.159 | 2.833 | 0.95-8.47 | 0.062 |
| Age ≤48 | 2.027 | 0.87-4.75 | 0.104 | 5.200 | 0.53-51.49 | 0.159 | 2.292 | 0.85-6.17 | 0.100 |
| Age ≤49 | 1.758 | 0.77-4.03 | 0.183 | 5.200 | 0.53-51.49 | 0.159 | 1.960 | 0.74-5.17 | 0.174 |
| Age ≤50 | 1.627 | 0.74-3.57 | 0.225 | 2.567 | 0.41-15.95 | 0.312 | 2.007 | 0.80-5.03 | 0.138 |
| Age ≤64 | 1.196 | 0.73-1.95 | 0.475 | 2.389 | 1.09-5.26 | 0.030 | 1.004 | 0.51-1.98 | 0.992 |
| Age 65-74 | 0.979 | 0.60-1.60 | 0.933 | 1.026 | 0.50-2.10 | 0.945 | 0.766 | 0.38-1.57 | 0.464 |
| Age ≥75 | 0.756 | 0.39-1.48 | 0.415 | 0.283 | 0.10-0.81 | 0.018 | 1.580 | 0.65-3.87 | 0.317 |
| Age ≥80 | 1.286 | 0.53-3.09 | 0.575 | 0.447 | 0.09-2.25 | 0.329 | 2.533 | 0.86-7.43 | 0.090 |
| *Education degree* | | | | | | | | | |
| Elementary | 0.654 | 0.30-1.42 | 0.284 | 0.788 | 0.31-2.01 | 0.617 | 0.184 | 0.02-1.41 | 0.103 |
| High school | 1.457 | 0.90-2.37 | 0.130 | 1.173 | 0.57-2.40 | 0.664 | 1.839 | 0.92-3.69 | 0.086 |
| College | 1.638 | 0.85-3.14 | 0.138 | 1.795 | 0.66-4.90 | 0.253 | 1.580 | 0.65-3.87 | 0.317 |
| University | 0.487 | 0.25-0.94 | 0.031 | 0.563 | 0.20-1.55 | 0.267 | 0.504 | 0.21-1.22 | 0.128 |
| *Employment status* | | | | | | | | | |
| Employed  Unemployed  Retired | 0.807 | 0.46-1.43 | 0.465 | 2.433 | 0.79-7.51 | 0.122 | 0.737 | 0.35-1.55 | 0.421 |
|  | 1.470 | 0.60-3.60 | 0.399 | 0.686 | 0.17-2.79 | 0.598 | 2.601 | 0.81-8.41 | 0.110 |
|  | 1.060 | 0.63-1.79 | 0.828 | 0.663 | 0.27-1.63 | 0.369 | 0.985 | 0.49-1.97 | 0.967 |
| *Marital status* | | | | | | | | | |
| Married/living with a partner | 0.624 | 0.36-1.07 | 0.087 | 0.727 | 0.34-1.55 | 0.409 | 0.698 | 0.31-1.58 | 0.387 |
| Alone/divorced | 1.700 | 0.79-3.64 | 0.172 | 2.180 | 0.56-8.56 | 0.264 | 1.825 | 0.70-4.78 | 0.220 |
| Widow(er) | 1.363 | 0.71-2.62 | 0.353 | 1.096 | 0.49-2.47 | 0.826 | 0.882 | 0.24-3.25 | 0.850 |
| *Cigarette smoking* | | | | | | | | | |
| Smoker  Former smoker  Non-smoker | 0.961 | 0.48-1.91 | 0.910 | 0.904 | 0.28-2.88 | 0.865 | 1.176 | 0.49-2.82 | 0.716 |
|  | 1.055 | 0.62-1.80 | 0.845 | 1.015 | 0.39-2.66 | 0.975 | 1.500 | 0.75-3.00 | 0.251 |
|  | 0.976 | 0.60-1.59 | 0.923 | 1.039 | 0.47-2.32 | 0.925 | 0.615 | 0.31-1.23 | 0.171 |
| *Functional mobility* | | | | | | | | | |
| Fully mobile  Mobile with help  Immobile | 0.567 | 0.21-1.51 | 0.257 | 0.743 | 0.19-2.92 | 0.670 | 0.500 | 0.12-2.09 | 0.342 |
|  | 1.763 | 0.66-4.70 | 0.257 | 1.345 | 0.34-5.28 | 0.670 | 2.000 | 0.48-8.35 | 0.342 |
|  | ----- | ----- | ----- | ----- | ----- | ----- | ----- | ----- | ----- |
| *AF characteristics* | | | | | | | | | |
| Total AF history (years) | 1.022 | 0.99-1.06 | 0.222 | 1.048 | 0.98-1.12 | 0.161 | 1.022 | 0.98-1.07 | 0.339 |
| Permanent AF | 0.973 | 0.57-1.66 | 0.919 | 0.606 | 0.27-1.38 | 0.232 | 1.444 | 0.70-2.96 | 0.316 |
| *Comorbid conditions* | | | | | | | | | |
| Hypertension | 0.766 | 0.42-1.41 | 0.390 | 0.327 | 0.12-0.91 | 0.033 | 1.129 | 0.48-2.66 | 0.782 |
| Heart failure | 0.960 | 0.43-2.14 | 0.921 | 1.345 | 0.34-5.28 | 0.670 | 0.959 | 0.34-2.73 | 0.938 |
| LVEF <50% | 0.721 | 0.35-1.48 | 0.372 | 0.315 | 0.04-2.78 | 0.298 | 1.176 | 0.53-2.63 | 0.692 |
| *Ischemic heart disease* | 0.575 | 0.28-1.17 | 0.125 | 0.221 | 0.05-1.03 | 0.054 | 1.3027 | 0.45-2.35 | 0.950 |
| Recent ACS | 2.697 | 0.17-43.57 | 0.485 | ----- | ----- | 1.000 | 0.000 | 0.00----- | 1.000 |
| Prior MI | 0.532 | 0.20-1.44 | 0.214 | 0.000 | 0.00----- | 0.999 | 0.907 | 0.32-2.57 | 0.855 |
| Chronic stable CAD | 0.369 | 0.08-1.66 | 0.193 | 0.643 | 0.12-3.46 | 0.607 | 0.000 | 0.00---- | 0.999 |
| PCI/Balloon angioplasty | 0.799 | 0.33-1.93 | 0.619 | 0.000 | 0.00---- | 0.999 | 1.706 | 0.66-4.43 | 0.273 |
| CABG | 0.890 | 0.18-4.49 | 0.888 | ----- | ------ | ----- | 1.300 | 0.25-6.69 | 0.754 |
| Cardiomyopathy | 0.799 | 0.33-1.93 | 0.619 | 0.643 | 0.12-3.46 | 0.607 | 1.081 | 0.38-3.10 | 0.885 |
| Valvular disease | 1.562 | 0.66-3.68 | 0.306 | 0.986 | 0.33-2.91 | 0.979 | 2.000 | 0.48-8.35 | 0.342 |
| Supraventricular arrhythmias (SA+AFL) | 0.367 | 0.16-0.85 | 0.019 | 0.590 | 0.20-1.78 | 0.348 | 0.203 | 0.05-0.89 | 0.034 |
| Ventricular arrhythmias | 0.799 | 0.33-1.93 | 0.619 | 2.180 | 0.56-8.56 | 0.264 | 0.376 | 0.08-1.68 | 0.201 |
| *CIEDs* | 2.258 | 0.98-5.18 | 0.055 | 2.180 | 0.56-8.56 | 0.264 | 2.533 | 0.86-7.43 | 0.090 |
| Anti-bradycardia pacemaker | 2.567 | 10.1-6.54 | 0.048 | 2.303 | 0.49-10.77 | 0.289 | 2.992 | 0.90-9.96 | 0.074 |
| ICD | 0.000 | 0.00---- | 0.999 | 0.000 | 0.00---- | 1.000 | 0.000 | 0.00---- | 1.000 |
| CRT | 2.716 | 0.38-19.58 | 0.321 | ---- | ----- | 1.000 | 1.991 | 0.17-22.05 | 0.589 |
| Peripheral artery disease | 0.921 | 0.09-8.68 | 0.921 | 1.660 | 0.10-27.16 | 0.722 | 0.000 | 0.00--- | 0.999 |
| Diabetes mellitus type II | 1.401 | 0.78-2.53 | 0.262 | 0.960 | 0.40-2.31 | 0.927 | 1.856 | 0.83-4.15 | 0.132 |
| Prior stroke/TIA | 0.797 | 0.21-2.96 | 0.734 | 1.250 | 0.27-5.84 | 0.777 | 0.000 | 0.00--- | 0.999 |
| Chronic kidney disease | 0.884 | 0.36-2.16 | 0.786 | 1.750 | 0.41-7.16 | 0.466 | 0.656 | 0.18-2.35 | 0.518 |
| COPD | 2.027 | 0.87-4.75 | 0.104 | 2.180 | 0.56-8.56 | 0.264 | 2.054 | 0.66-6.37 | 0.213 |
| Malignancy | 1.948 | 0.72-5.29 | 0.190 | 1.250 | 0.27-5.84 | 0.777 | 2.737 | 0.74-10.18 | 0.133 |
| Thyroid dysfunction | 0.997 | 0.55-1.80 | 0.992 | 0.641 | 0.27-1.50 | 0.304 | 1.305 | 0.56-3.03 | 0.535 |
| Hyperlipoproteinemia | 1.070 | 0.64-1.78 | 0.794 | 0.828 | 0.39-1.78 | 0.628 | 1.360 | 0.68-2.73 | 0.387 |
| Other conditions | 1.272 | 0.61-2.64 | 0.519 | 1.469 | 0.46-4.66 | 0.514 | 1.254 | 0.47-3.37 | 0.653 |
| CHA_2_DS_2_-VASc score | 1.010 | 0.56-1.19 | 0.905 | 0.636 | 0.46-0.88 | 0.006 | 1.061 | 0.84-1.34 | 0.615 |
| >1 non-sex CHA_2_DS_2_-VASc risk factors | 1.081 | 0.47-2.51 | 0.856 | 0.110 | 0.01-0.97 | 0.047 | 2.043 | 0.58-7.18 | 0.265 |
| *Current medication* | | | | | | | | | |
| OAC | 0.950 | 0.42-2.14 | 0.901 | 0.789 | 0.26-2.43 | 0.679 | 1.327 | 0.37-4.81 | 0.667 |
| VKA | 1.736 | 10.5-2.88 | 0.033 | 1.870 | 0.89-3.91 | 0.096 | 1.785 | 0.87-3.68 | 0.117 |
| NOAC | 0.520 | 0.30-0.91 | 0.021 | 0.437 | 0.19-0.98 | 0.045 | 0.576 | 0.26-1.26 | 0.165 |
| OAC treatment duration (years) | 1.060 | 0.99-1.13 | 0.076 | 1.107 | 0.99-1.24 | 0.085 | 1.052 | 0.97-1.14 | 0.216 |
| OAC treatment duration less than 1 year | 0.499 | 0.27-0.92 | 0.025 | 0.505 | 0.21-1.20 | 0.121 | 0.433 | 0.18-1.05 | 0.064 |
| ASA | 1.080 | 0.50-2.35 | 0.846 | 0.627 | 0.19-2.12 | 0.454 | 1.633 | 0.59-4.51 | 0.343 |
| P2Y_12_ inhibitor | 1.140 | 0.48-2.71 | 0.766 | 0.529 | 0.10-2.73 | 0.447 | 1.910 | 0.68-5.37 | 0.220 |
| Beta blocker | 0.710 | 0.40-1.27 | 0.246 | 0.523 | 0.22-1.22 | 0.135 | 1.006 | 0.42-2.38 | 0.990 |
| Non-DHP Ca blocker | 2.756 | 0.67-11.26 | 0.158 | 3.391 | 0.30-38.44 | 0.324 | 2.650 | 0.43-16.40 | 0.295 |
| Digitalis | 2.080 | 0.70-6.17 | 0.187 | 1.101 | 0.18-6.84 | 0.917 | 3.305 | 0.85-12.90 | 0.085 |
| Antiarrhythmic drugs | 1.134 | 0.69-1.86 | 0.619 | 1.435 | 0.68-3.03 | 0.344 | 0.876 | 0.44-1.73 | 0.703 |
| Mexiletine | ---- | ---- | ---- | ---- | ---- | ----- | ----- | ----- | ---- |
| Propafenone | 0.881 | 0.40-1.95 | 0.755 | 0.802 | 0.26-2.51 | 0.705 | 0.898 | 0.29-2.83 | 0.854 |
| Flecainide | 0.932 | 0.38-2.29 | 0.878 | 1.705 | 0.41-7.16 | 0.466 | 0.702 | 0.20-2.53 | 0.589 |
| Sotalol | 5.455 | 0.49-60.90 | 0.168 | 1.660 | 0.10-27.16 | 0.722 | ---- | ---- | 1.000 |
| Amiodarone | 1.131 | 0.69-1.85 | 0.626 | 1.310 | 0.63-2.71 | 0.465 | 0.920 | 0.45-1.87 | 0.817 |
| DHP Ca blocker | 0.746 | 0.44-1.26 | 0.275 | 0.466 | 0.22-0.99 | 0.047 | 0.895 | 0.42-1.93 | 0.777 |
| ACEI/ARB | 0.925 | 0.55-1.56 | 0.770 | 0.643 | 0.30-1.38 | 0.258 | 1.388 | 0.63-3.04 | 0.414 |
| Diuretic | 0.644 | 0.40-1.05 | 0.077 | 0.319 | 0.15-0.68 | 0.003 | 1.018 | 0.52-2.01 | 0.959 |
| Spironolactone | 0.998 | 0.57-1.75 | 0.994 | 1.692 | 0.70-4.09 | 0.243 | 0.788 | 0.36-1.73 | 0.553 |
| Statins | 1.309 | 0.80-2.13 | 0.281 | 1.064 | 0.52-2.19 | 0.867 | 1.442 | 0.73-2.87 | 0.296 |
| Sedative | 1.259 | 0.59-2.69 | 0.552 | 1.469 | 0.46-4.66 | 0.514 | 1.153 | 0.40-3.33 | 0.793 |
| PPI | 1.792 | 1.07-3.00 | 0.027 | 2.786 | 1.23-6.34 | 0.015 | 1.506 | 0.74-3.06 | 0.258 |
| Insulin | 2.388 | 0.78-7.31 | 0.127 | 1.705 | 0.41-7.16 | 0.466 | 2.650 | 0.43-16.40 | 0.295 |
| Oral antidiabetic drug | 1.316 | 0.69-2.52 | 0.408 | 0.929 | 0.36-2.41 | 0.879 | 1.671 | 0.68-4.11 | 0.264 |
| Other medications | 1.821 | 1.11-2.98 | 0.017 | 1.548 | 0.75-3.19 | 0.237 | 1.923 | 0.97-3.83 | 0.063 |
| *Non-pharmacological treatment* | | | | | | | | | |
| Ablation/ECV | 0.519 | 0.31-0.87 | 0.013 | 1.042 | 0.48-2.26 | 0.918 | 0.362 | 0.17-0.76 | 0.007 |
| ECV AF | 0.435 | 0.24-0.80 | 0.007 | 0.623 | 0.24-1.63 | 0.336 | 0.429 | 0.19-0.96 | 0.038 |
| ECV AFL | 0.000 | 0.00--- | 0.999 | 0.000 | 0.00--- | 0.999 | 0.000 | 0.00--- | 0.999 |
| AF Ablation | 0.800 | 0.41-1.57 | 0.517 | 1.795 | 0.66-4.90 | 0.253 | 0.411 | 0.14-1.24 | 0.113 |
| AFL Ablation | 0.000 | 0.00---- | 0.999 | ---- | ----- | ----- | 0.000 | 0.00---- | 0.999 |
| Ablation other arrhythmias | 2.697 | 0.17-43.57 | 0.485 | ----- | ----- | 1.000 | 0.000 | 0.000 | 1.000 |
| *Multimorbidity and polypharmacy* | | | | | | | | | |
| Polypharmacy | 1.126 | 0.65-1.94 | 0.669 | 0.794 | 0.35-1.81 | 0.585 | 1.332 | 0.62-2.86 | 0.461 |
| N of drugs | 1.061 | 0.97-1.16 | 0.179 | 0.978 | 0.85-1.12 | 0.744 | 1.120 | 0.996-1.26 | 0.059 |
| N of pills | 1.045 | 0.97-1.12 | 0.237 | 0.987 | 0.88-1.11 | 0.835 | 1.080 | 0.98-1.20 | 0.114 |
| N of drugs without OAC | 1.062 | 0.97-1.16 | 0.193 | 0.975 | 0.85-1.12 | 0.720 | 1.125 | 0.99-1.27 | 0.062 |
| N of pills without OAC | 1.047 | 0.97-1.13 | 0.224 | 0.990 | 0.88-1.12 | 0.867 | 1.082 | 0.98-1.19 | 0.114 |
| Parenteral drug use | 2.174 | 0.79-6.02 | 0.135 | 1.721 | 0.47-6.29 | 0.411 | 1.975 | 0.35-11.17 | 0.441 |
| N of parenteral applications daily | 1.272 | 0.88-1.85 | 0.207 | 1340 | 0.83-2.15 | 0.227 | 0.851 | 0.34-2.12 | 0.730 |
| N of comorbidities | 0.961 | 0.84-1.11 | 0.584 | 0.872 | 0.70-1.09 | 0.231 | 1.033 | 0.85-1.25 | 0.738 |
| Patients with multimorbidity (without SA/ VA) | 0.929 | 0.35-2.47 | 0.883 | 0.000 | 0.00---- | 0.999 | 4.184 | 0.54-32.62 | 0.172 |

**AF:** Atrial fibrillation; **LVEF:** Left ventricular ejection fraction; **ACS:** Acute coronary syndrome; **MI:** Myocardial infarction; **CAD:** Coronary artery disease; **PCI:** Percutaneous coronary intervention; **CABG:** Coronary artery bypass grafting; **AFL:** Atrial flutter; **CIED:** Cardiac implantable electronic devices; **ICD:** Implantable cardioverter defibrillator; **CRT:** Cardiac resynchronisation therapy; **TIA:** Transient ischemic attack, **COPD:** Chronic obstructive pulmonary disease; **OAC:** Oral anticoagulant therapy; **VKA:** Vitamin K antagonist; **NOAC:** Non-vitamin K antagonist oral anticoagulant; **ASA:** Acetylsalicylic acid; **DHP:** Dihydropyridine; **ACEI:** Angiotensin converting enzyme inhibitor; **ARB:** Angiotensin receptor inhibitor; **PPI:** Proton pump inhibitor; **ECV:** electrical cardioversion; **SA:** Supraventricular arrhythmias; **VA:** Ventricular arrhythmias; **N:** Number.

**Table S5. Univariate analyses of the lowest Treatment Burden Questionnaire score in patients with atrial fibrillation**

| **Variable** | **TBQ ≤26 points**  **Patients with AF**  **N=73 (%)** | | | **TBQ ≤26 points**  **Females**  **n=21 (28.8)** | | | **TBQ ≤26points**  **Males**  **N=52 (71.2)** | | |
| --- | --- | --- | --- | --- | --- | --- | --- | --- | --- |
|  | **OR** | **CI 95%** | **P value** | **OR** | **CI 95%** | **P value** | **OR** | **CI 95%** | **P value** |
| Age | 1.024 | 0.997-1.05 | 0.083 | 1.026 | 0.97-1.09 | 0.387 | 1.031 | 1.00-1.06 | 0.054 |
| Age ≤40 | 0.000 | 0.00--- | 0.999 | 0.000 | 0.00--- | 1.000 | 0.000 | 0.00--- | 0.999 |
| Age ≤45 | 0.242 | 0.03-1.87 | 0.174 | 0.000 | 0.00--- | 0.999 | 0.251 | 0.03-2.00 | 0.191 |
| Age ≤47 | 0.185 | 0.02-1.41 | 0.104 | 0.000 | 0.00--- | 0.999 | 0.193 | 0.03-1.51 | 0.117 |
| Age ≤48 | 0.302 | 0.07-1.32 | 0.111 | 0.000 | 0.00--- | 0.999 | 0.298 | 0.07-1.33 | 0.113 |
| Age ≤49 | 0.275 | 0.06-1.19 | 0.084 | 0.000 | 0.00--- | 0.999 | 0.264 | 0.06-1.17 | 0.080 |
| Age ≤50 | 0.231 | 0.05-0.995 | 0.049 | 0.000 | 0.00--- | 0.999 | 0.224 | 0.05-0.99 | 0.048 |
| Age ≤64 | 0.723 | 0.42-1.25 | 0.242 | 0.544 | 0.17-1.75 | 0.306 | 0.677 | 0.36-1.28 | 0.232 |
| Age 65-74 | 1.321 | 0.78-2.23 | 0.296 | 1.142 | 0.45-2.92 | 0.781 | 1.556 | 0.82-2.95 | 0.175 |
| Age ≥75 | 1.054 | 0.53-2.08 | 0.880 | 1.527 | 0.53-4.39 | 0.432 | 0.919 | 0.37-2.30 | 0.857 |
| Age ≥80 | 1.126 | 0.43-2.93 | 0.807 | 0.613 | 0.07-5.17 | 0.653 | 1.364 | 0.45-4.13 | 0.583 |
| *Education degree* | | | | | | | | | |
| Elementary | 1.803 | 0.90-3.62 | 0.097 | 1.431 | 0.47-4.39 | 0.531 | 2.683 | 1.04-6.90 | 0.041 |
| High school | 0.907 | 0.54-1.53 | 0.713 | 0.722 | 0.28-1.86 | 0.499 | 1.000 | 0.53-1.88 | 1.000 |
| College | 0.691 | 0.31-1.55 | 0.371 | 0.262 | 0.03-2.08 | 0.205 | 0.919 | 0.37-2.30 | 0.857 |
| University | 0.947 | 0.51-1.77 | 0.864 | 2.250 | 0.76-6.67 | 0.143 | 0.604 | 0.28-1.31 | 0.201 |
| *Employment status* | | | | | | | | | |
| Employed  Unemployed  Retired | 0.723 | 0.38-1.36 | 0.313 | 0.358 | 0.04-2.89 | 0.335 | 0.650 | 0.32-1.30 | 0.225 |
|  | 0.510 | 0.15-1.77 | 0.288 | 0.539 | 0.07-4.50 | 0.568 | 0.513 | 0.11-2.39 | 0.395 |
|  | 1.582 | 0.88-2.86 | 0.128 | 2.488 | 0.54-11.50 | 0.243 | 1.727 | 0.88-3.38 | 0.110 |
| *Marital status* | | | | | | | | | |
| Married/living with a partner | 1.200 | 0.64-2.23 | 0.566 | 1.716 | 0.58-5.06 | 0.327 | 0.842 | 0.39-1.84 | 0.666 |
| Alone/divorced | 1.200 | 0.52-2.80 | 0.673 | 0.613 | 0.07-5.17 | 0.653 | 1.322 | 0.51-3.42 | 0.564 |
| Widow(er) | 0.651 | 0.29-1.46 | 0.298 | 0.625 | 0.19-2.01 | 0.431 | 0.972 | 0.30-3.16 | 0.963 |
| *Cigarette smoking* | | | | | | | | | |
| Smoker  Former smoker  Non-smoker | 0.766 | 0.35-1.66 | 0.501 | 0.358 | 0.04-2.89 | 0.335 | 0.842 | 0.36-1.99 | 0.695 |
|  | 1.455 | 0.83-2.54 | 0.187 | 3.286 | 1.13-9.55 | 0.029 | 0.961 | 0.50-1.86 | 0.906 |
|  | 0.826 | 0.49-1.39 | 0.473 | 0.555 | 0.21-1.48 | 0.241 | 1.141 | 0.61-2.14 | 0.682 |
| *Functional mobility* | | | | | | | | | |
| Fully mobile  Mobile with help  Immobile | 1.440 | 0.41-5.12 | 0.573 | 1.633 | 0.19-13.79 | 0.653 | 1.207 | 0.24-6.00 | 0.818 |
|  | 0.694 | 0.20-2.47 | 0.573 | 0.613 | 0.07-5.17 | 0.653 | 0.829 | 0.17-4.12 | 0.818 |
|  | ----- | ----- | ----- | ----- | ----- | ----- | ----- | ----- | ----- |
| *AF characteristics* | | | | | | | | | |
| Total AF history (years) | 0.976 | 0.93-1.02 | 0.286 | 1.036 | 0.96-1.12 | 0.376 | 0.948 | 0.90-1.00 | 0.066 |
| Permanent AF | 0.400 | 0.21-0.78 | 0.008 | 0.353 | 0.10-1.28 | 0.113 | 0.415 | 0.19-0.92 | 0.030 |
| *Comorbid conditions* | | | | | | | | | |
| Hypertension | 0.914 | 0.47-1.78 | 0.792 | 1.689 | 0.36-7.97 | 0.508 | 0.820 | 0.38-1.75 | 0.607 |
| Heart failure | 1.813 | 0.84-3.92 | 0.131 | 2.778 | 0.64-12.13 | 0.174 | 1.444 | 0.58-3.58 | 0.427 |
| LVEF <50% | 1.849 | 0.95-3.58 | 0.069 | 1.010 | 0.11-9.12 | 0.993 | 1.719 | 0.83-3.55 | 0.142 |
| *Ischemic heart disease* | 1.607 | 085-3.04 | 0.145 | 2.032 | 0.58-7.13 | 0.268 | 1.356 | 0.64-2.85 | 0.423 |
| Recent ACS | 0.000 | 0.00--- | 0.999 | 0.000 | 0.00--- | 1.000 | 0.000 | 0.00--- | 1.000 |
| Prior MI | 1.997 | 0.88-4.51 | 0.096 | 11.053 | 0.95-128.04 | 0.055 | 1.354 | 0.55-3.33 | 0.510 |
| Chronic stable CAD | 1.651 | 0.56-4.91 | 0.368 | 0.833 | 0.10-7.30 | 0.869 | 2.450 | 0.63-9.49 | 0.195 |
| PCI/Balloon angioplasty | 2.323 | 1.01-4.93 | 0.047 | 12.235 | 2.08-72.06 | 0.006 | 1.235 | 0.48-3.17 | 0.660 |
| CABG | 2.169 | 0.51-9.30 | 0.297 | ---- | ----- | ------- | 1.800 | 0.42-7.81 | 0.432 |
| Cardiomyopathy | 1.320 | 0.56-3.10 | 0.524 | 0.833 | 0.10-7.30 | 0.869 | 1.322 | 0.51-3.42 | 0.564 |
| Valvular disease | 1.414 | 0.57-3.53 | 0.458 | 1.192 | 0.31-4.61 | 0.799 | 2.450 | 0.63-9.49 | 0.195 |
| Supraventricular arrhythmias (SA+AFL) | 1.934 | 1.01-3.70 | 0.046 | 4.318 | 1.44-12.99 | 0.009 | 1.270 | 0.56-2.87 | 0.566 |
| Ventricular arrhythmias | 1.084 | 0.45-2.64 | 0.859 | 0.613 | 0.07-5.17 | 0.653 | 1.191 | 0.44-3.25 | 0.7333 |
| *CIEDs* | 0.654 | 0.22-1.97 | 0.451 | 0.000 | 0.00---- | 0.999 | 0.972 | 0.30-3.16 | 0.963 |
| Anti-bradycardia pacemaker | 0.185 | 0.02-1.41 | 0.104 | 0.000 | 0.00---- | 0.099 | 0.251 | 0.03-2.00 | 0.191 |
| ICD | 3.569 | 0.22-57.77 | 0.370 | 0.000 | 0.00---- | 1.000 | ----- | 0.00--- | 1.000 |
| CRT | 3.606 | 0.50-26.05 | 0.204 | 0.000 | 0.00---- | 1.000 | 6.040 | 0.54-68.04 | 0.146 |
| Peripheral artery disease | 1.181 | 0.12-11.52 | 0.886 | 0.000 | 0.00--- | 0.999 | 2.961 | 0.18-48.20 | 0.446 |
| Diabetes mellitus type II | 0.519 | 0.24-1.11 | 0.090 | 0.849 | 0.26-2.77 | 0.786 | 0.399 | 0.15-1.09 | 0.072 |
| Prior stroke/TIA | 1.063 | 0.29-3.97 | 0.928 | 0.833 | 0.10-7.30 | 0.869 | 1.480 | 0.26-8.33 | 0.656 |
| Chronic kidney disease | 0.961 | 0.37-2.47 | 0.933 | 0.000 | 0.00--- | 0.999 | 1.286 | 0.47-3.54 | 0.627 |
| COPD | 0.925 | 0.3-2.57 | 0.881 | 1.489 | 0.29-7.72 | 0.636 | 0.714 | 0.19-2.64 | 0.614 |
| Malignancy | 0.747 | 0.21-2.67 | 0.654 | 0.833 | 0.10-7.30 | 0.869 | 0.720 | 0.15-3.50 | 0.684 |
| Thyroid dysfunction | 0.955 | 0.50-1.81 | 0.887 | 1.538 | 0.56-4.22 | 0.403 | 0.771 | 0.33-1.81 | 0.551 |
| Hyperlipoproteinemia | 1.154 | 0.67-1.98 | 0.604 | 1.588 | 0.61-4.13 | 0.343 | 0.989 | 0.51-1.92 | 0.977 |
| Other conditions | 0.777 | 0.33-1.84 | 0.567 | 0.392 | 0.05-3.19 | 0.381 | 0.913 | 0.34-2.43 | 0.855 |
| CHA_2_DS_2_-VASc score | 1.017 | 0.86-1.21 | 0.850 | 1.246 | 0.86-1.81 | 0.247 | 1.058 | 0.85-1.31 | 0.611 |
| >1 non-sex CHA_2_DS_2_-VASc risk factors | 1.198 | 0.47-304 | 0.704 | ---- | 0.00---- | 0.999 | 1.095 | 0.41-2.91 | 0.855 |
| *Current medication* | | | | | | | | | |
| OAC | 0.833 | 0.36-1.94 | 0.673 | 0.695 | 0.18-2.74 | 0.603 | 0.879 | 0.30-1.60 | 0.816 |
| VKA | 0.769 | 0.46-1.30 | 0.325 | 0.533 | 0.22-1.43 | 0.220 | 0.869 | 0.46-1.64 | 0.644 |
| NOAC | 1.238 | 0.72-2.13 | 0.441 | 1.588 | 0.61-4.13 | 0.220 | 0.869 | 0.46-1.64 | 0.664 |
| OAC treatment duration (years) | 0.933 | 0.86-1.02 | 0.115 | 0.848 | 0.68-1.06 | 0.140 | 0.948 | 0.86-1.04 | 0.258 |
| OAC treatment duration less than 1 year | 1.620 | 0.91-2.88 | 0.099 | 1.807 | 0.63-5.15 | 0.268 | 1.633 | 0.81-3.28 | 0.167 |
| ASA | 0.871 | 0.36-2.08 | 0.757 | 1.439 | 0.37-5.68 | 0.603 | 0.662 | 0.21-2.07 | 0.477 |
| P2Y_12_ inhibitor | 0.789 | 0.29-2.16 | 0.644 | 0.707 | 0.08-6.07 | 0.752 | 0.761 | 0.24-2.41 | 0.642 |
| Beta blocker | 1.563 | 0.77-3.17 | 0.215 | 1.244 | 0.38-4.05 | 0.717 | 1.714 | 0.71-4.16 | 0.233 |
| Non-DHP Ca blocker | 0.000 | 0.00---- | 0.999 | 0.000 | 0.00---- | 0.999 | 0.000 | 0.00---- | 0.999 |
| Digitalis | 0.262 | 0.03-2.04 | 0.200 | 0.000 | 0.00---- | 0.999 | 0.353 | 0.04-2.89 | 0.322 |
| Antiarrhythmic drugs | 1.246 | 0.73-2.13 | 0.420 | 2.271 | 0.77-6.66 | 0.135 | 1.019 | 0.54-1.93 | 0.954 |
| Mexiletine | ---- | ---- | ---- | ---- | ---- | ---- | ---- | ---- | ---- |
| Propafenone | 1.416 | 0.65-3.09 | 0.382 | 3.000 | 0.91-.9.93 | 0.072 | 0.904 | 0.31-2.60 | 0.852 |
| Flecainide | 1.011 | 0.39-2.61 | 0.982 | 0.707 | 0.08-6.07 | 0.752 | 1.049 | 0.36-3.07 | 0.931 |
| Sotalol | 0.000 | 0.00---- | 0.999 | 0.000 | 0.00---- | 0.999 | 0.000 | 0.00---- | 0.999 |
| Amiodarone | 1.121 | 0.66-1.90 | 0.674 | 1.332 | 0.52-3.41 | 0.550 | 1.071 | 0.56-2.05 | 0.835 |
| DHP Ca blocker | 0.693 | 0.39-1.23 | 0.210 | 1.281 | 0.50-3.28 | 0605 | 0.532 | 0.25-1.15 | 0.108 |
| ACEI/ARB | 0.908 | 0.52-1.59 | 0.736 | 0.904 | 0.33-2.45 | 0.843 | 0.888 | 0.45-1.76 | 0.734 |
| Diuretic | 1.292 | 0.76-2.20 | 0.344 | 1.774 | 0.64-4.93 | 0.272 | 1.195 | 0.63-2.26 | 0.582 |
| Spironolactone | 1.219 | 0.68-2.20 | 0.511 | 0.952 | 0.29-3.13 | 0.936 | 1.244 | 0.62-2.48 | 0.535 |
| Statins | 0.756 | 0.44-1.30 | 0.310 | 0.906 | 0.35-2.33 | 0.838 | 0.725 | 0.37-1.41 | 0.342 |
| Sedative | 0.707 | 0.28-1.78 | 0.461 | 1.600 | 0.40-6.39 | 0.506 | 0.429 | 0.12-1.51 | 0.188 |
| PPI | 0.921 | 0.52-1.65 | 0.780 | 1.231 | 0.43-3.50 | 0.697 | 0.774 | 0.38-1.56 | 0.188 |
| Insulin | 0.285 | 0.04-2.23 | 0.231 | 0.000 | 0.00---- | 0.999 | 0.725 | 0.08-6.64 | 0.776 |
| Oral antidiabetic drug | 0.436 | 0.18-1.07 | 0.069 | 0.763 | 0.20-2.85 | 0.688 | 0.311 | 0.09-1.08 | 0.065 |
| Other medications | 0.860 | 0.50-1.48 | 0.584 | 0.978 | 0.38-2.52 | 0.964 | 0.857 | 0.44-1.67 | 0.649 |
| *Non-pharmacological treatment* | | | | | | | | | |
| Ablation/ECV | 1.156 | 0.68-1.95 | 0.589 | 0.662 | 0.22-1.96 | 0.455 | 1.265 | 0.67-2.38 | 0.465 |
| ECV AF | 1.265 | 0.72-2.21 | 0.409 | 1.431 | 0.47-4.39 | 0.531 | 1.075 | 0.56-2.07 | 0.828 |
| ECV AFL | 0.000 | 0.00---- | 0.999 | 0.000 | 0.00---- | 0.999 | 0.000 | 0.00---- | 0.999 |
| AF Ablation | 1.576 | 0.82-3.03 | 0.171 | 0.262 | 0.03-2.08 | 0.205 | 2.396 | 1.13-5.08 | 0.023 |
| AFL Ablation | 4.669 | 1.22-17.86 | 0.024 | ----- | ----- | ------ | 3.936 | 1.02-15.26 | 0.048 |
| Ablation other arrhythmias | 0.000 | 0.00---- | 0.999 | 0.000 | 0.00---- | 1.000 | 0.000 | 0.00---- | 1.000 |
| *Multimorbidity and polypharmacy* | | | | | | | | | |
| Polypharmacy | 0.897 | 0.51-1.59 | 0.709 | 2.154 | 0.59-7.87 | 0.246 | 0.708 | 0.36-1.38 | 0.308 |
| N of drugs | 0.952 | 0.86-1.05 | 0.323 | 1.050 | 0.89-1.25 | 0.579 | 0.917 | 0.81-1.03 | 0.157 |
| N of pills | 0.981 | 0.91-1.06 | 0.634 | 1.080 | 0.93-1.25 | 0.314 | 0.948 | 0.86-1.05 | 0.283 |
| N of drugs without OAC | 0.955 | 0.86-1.06 | 0.373 | 1.071 | 0.90-1.28 | 0.445 | 0.911 | 0.80-1.03 | 0.150 |
| N of pills without OAC | 0.981 | 0.90-1.07 | 0.656 | 1.085 | 0.93-1.26 | 0.285 | 0.946 | 0.86-1.05 | 0.281 |
| Parenteral drug use | 0.491 | 0.11-2.21 | 0.354 | 0.539 | 0.07-4.50 | 0.568 | 0.576 | 0.07-5.05 | 0.619 |
| N of parenteral applications daily | 0.781 | 0.44-1.39 | 0.397 | 0.727 | 0.30-1.78 | 0.484 | 0.921 | 0.43-1.99 | 0.835 |
| N of comorbidities | 1.074 | 0.93-1.24 | 0.327 | 1.133 | 0.89-1.44 | 0.303 | 1.047 | 0.88-1.25 | 0.609 |
| Patients with multimorbidity (without SA/ VA) | 0.899 | 0.32-2.54 | 0.841 | ----- | 0.00--- | 0.999 | 0.733 | 0.24-2.22 | 0.583 |

**TBQ:** Treatment burden questionnaire**; AF:** Atrial fibrillation; **LVEF:** Left ventricular ejection fraction; **ACS:** Acute coronary syndrome; **MI:** Myocardial infarction; **CAD:** Coronary artery disease; **PCI:** Percutaneous coronary intervention; **CABG:** Coronary artery bypass grafting; **AFL:** Atrial flutter; **CIED:** Cardiac implantable electronic devices; **ICD:** Implantable cardioverter defibrillator; **CRT:** Cardiac resynchronisation therapy; **TIA:** Transient ischemic attack, **COPD:** Chronic obstructive pulmonary disease; **OAC:** Oral anticoagulant therapy; **VKA:** Vitamin K antagonist; **NOAC:** Non-vitamin K antagonist oral anticoagulant; **ASA:** Acetylsalicylic acid; **DHP:** Dihydropyridine; **ACEI:** Angiotensin converting enzyme inhibitor; **ARB:** Angiotensin receptor inhibitor; **PPI:** Proton pump inhibitor; **ECV:** electrical cardioversion; **SA:** Supraventricular arrhythmias; **VA:** Ventricular arrhythmias; **N:** Number.

**Table S6. Univariate analyses of QOL 5D as a continuous variable in patients with AF.**

| **Variable** | **AF patients**  **n=331 (%)** | | | **Female**  **n=127 (38.4)** | | | **Male**  **n=204 (61.6)** | | |
| --- | --- | --- | --- | --- | --- | --- | --- | --- | --- |
|  | **beta** | **CI 95%** | **P value** | **beta** | **CI 95%** | **P value** | **beta** | **CI 95%** | **P value** |
| Age | 0.241 | 0.04-0.11 | <0.001 | 0.194 | 0.01-0.14 | 0.029 | 0.211 | 0.02-0.10 | 0.002 |
| Age ≤40 | 0.028 | -1.82-3.08 | 0.613 | -0.081 | -9.51-3.52 | 0.365 | 0.095 | -0.79-4.24 | 0.177 |
| Age ≤45 | -0.046 | -2.41-0.97 | 0.403 | -0.142 | -6.81-0.72 | 0.112 | 0.021 | -1.54-2.09 | 0.764 |
| Age ≤47 | -0.073 | -2.52-0.50 | 0.188 | -0.164 | -6.33-0.20 | 0.065 | -0.005 | -1.70-1.57 | 0.941 |
| Age ≤48 | -0.111 | -2.74-(-0.04) | 0.044 | -0.164 | -6.33-0.20 | 0.065 | -0.056 | -2.01-0.85 | 0.425 |
| Age ≤49 | -0.124 | -2.80-(-0.20) | 0.024 | -0.164 | -6.33-0.20 | 0.065 | -0.073 | -2.09-0.65 | 0.302 |
| Age ≤50 | -0.132 | -2.71-(-0.27) | 0.017 | -0.172 | -5.81-0.04 | 0.053 | -0.078 | -2.03-0.56 | 0.266 |
| Age ≤64 | -0.180 | -1.90-(-0.49) | 0.001 | -0.021 | -1.43-1.13 | 0.817 | -0.212 | -.14-(-0.47) | 0.002 |
| Age 65-74 | 0.033 | -0.50-0.93 | 0.553 | -0.087 | -1.72-0.58 | 0.331 | 0.068 | -0.45-1.31 | 0.333 |
| Age ≥75 | 0.191 | 0.73-2.56 | <0.001 | 0.127 | -0.38-2.38 | 0.154 | 0.208 | 0.64-3.03 | 0.003 |
| Age ≥80 | 0.166 | 0.73-3.35 | 0.002 | 0.069 | -1.37-3.12 | 0.444 | 0.242 | 1.23-4.31 | <0.001 |
| *Education degree* | | | | | | | | | |
| Elementary | 0.121 | 0.12-2.18 | 0.028 | 0.122 | -0.45-2.48 | 0.173 | 0.067 | -0.74-2.13 | 0.340 |
| High school | -0.046 | -1.00-0.41 | 0.408 | -0.126 | -1.97-0.33 | 0.159 | 0.006 | -0.81-0.89 | 0.928 |
| College | -0.002 | -1.03-0.99 | 0.966 | 0.052 | -1.16-2.14 | 0.559 | -0.038 | -1.56-0.88 | 0.587 |
| University | -0.041 | -1.16-0.52 | 0.454 | -0.008 | -1.60-1.45 | 0.926 | -0.022 | -1.3-0.82 | 0.751 |
| *Employment status* | | | | | | | | | |
| Employed  Unemployed  Retired | -0.211 | -2.37-(-0.79) | <0.001 | -0.089 | -2.77-0.91 | 0.319 | -0.193 | -2.14-(-0.37) | 0.407 |
|  | -0.047 | -1.99-0.78 | 0.390 | -0.087 | -3.19-1.09 | 0.332 | -0.034 | -2.17-1.32 | 0.629 |
|  | 0.222 | 0.81-2.28 | <0.001 | 0.131 | -0.37-2.55 | 0.142 | 0.203 | 0.42-2.13 | 0.004 |
| *Marital status* | | | | | | | | | |
| Married/living with a partner | -0.112 | -1.66-(-0.03) | 0.042 | -0.099 | -1.91-0.53 | 0.268 | -0.058 | -1.54-0.63 | 0.407 |
| Alone/divorced | -0.062 | -1.87-0.51 | 0.264 | -0.026 | -2.58-1.93 | 0.776 | -0.058 | -1.91-0.79 | 0.413 |
| Widow(er) | 0.187 | 0.73-2.68 | 0.001 | 0.121 | -0.41-2.21 | 0.174 | 0.153 | 0.18-3.32 | 0.029 |
| *Cigarette smoking* | | | | | | | | | |
| Smoker  Former smoker  Non-smoker | 0.016 | -0.84-1.14 | 0.768 | -0.043 | -2.29-1.40 | 0.633 | 0.083 | -0.45-1.82 | 0.237 |
|  | 0.075 | -0.24-1.32 | 0.174 | 0.037 | -1.23-1.88 | 0.681 | 0.183 | 0.30-2.05 | 0.009 |
|  | -0.080 | -1.23-0.19 | 0.148 | -0.001 | -1.30-1.29 | 0.995 | -0.237 | -2.29-(-0.63) | 0.001 |
| *Functional mobility* | | | | | | | | | |
| Fully mobile  Mobile with help  Immobile | -3.19 | -6.04-(-3.10) | <0.001 | -0.285 | -5.78-(-1.46) | 0.001 | -0.342 | -7.08-(-3.17) | <0.001 |
|  | 0.319 | 3.10-6.04 | <0.001 | 0.285 | 1.46-5.78 | 0.001 | 0.342 | 3.17-7.08 | <0.001 |
|  | ----- | ----- | ----- | ----- | ----- | ----- | ----- | ----- | ----- |
| *AF characteristics* | | | | | | | | | |
| Total AF history (years) | 0.068 | -0.02-0.09 | 0.219 | 0.035 | -0.09-0.13 | 0.701 | 0.130 | -0.00-0.12 | 0.064 |
| Permanent AF | 0.031 | -0.55-1.00 | 0.568 | -0.063 | -1.72-0.82 | 0.483 | 0.097 | -0.28-1.58 | 0.170 |
| *Comorbid conditions* | | | | | | | | | |
| Hypertension | 0.133 | 0.22-2.03 | 0.015 | 0.224 | 0.49-3.71 | 0.011 | 0.058 | -0.62-1.49 | 0.414 |
| Heart failure | 0.225 | 1.28-3.54 | <0.001 | 0.003 | -2.22-2.29 | 0.976 | 0.389 | 2.44-4.84 | <0.001 |
| LVEF <50% | 0.118 | 0.09-2.04 | 0.032 | 0.082 | -1.46-3.97 | 0.361 | 0.232 | 0.72-2.74 | 0.001 |
| *Ischemic heart disease* | 0.060 | -0.41-1.44 | 0.274 | -0.064 | -2.43-1.14 | 0.476 | 0.176 | 0.30-2.36 | 0.012 |
| Recent ACS | 0.181 | 3.12-12.06 | 0.001 | 0.083 | -3.46-9.57 | 0.355 | 0.267 | 5.58-17.62 | <0.001 |
| Prior MI | -0.022 | -1.50-0.99 | 0.690 | -0.110 | -6.14-1.42 | 0.219 | 0.065 | -0.68-1.88 | 0.356 |
| Chronic stable CAD | 0.047 | -0.94-2.35 | 0.399 | 0.013 | -2.35-2.72 | 0.885 | 0.063 | -1.13-3.01 | 0.372 |
| PCI/Balloon angioplasty | 0.043 | -0.74-1.72 | 0.433 | -0.066 | -3.73-1.70 | 0.460 | 0.140 | 0.02-2.64 | 0.046 |
| CABG | 0.039 | -1.48-3.11 | 0.485 | ---- | ---- | ---- | 0.094 | -0.70-3.67 | 0.182 |
| Cardiomyopathy | 0.040 | -0.77-1.68 | 0.468 | 0.066 | -1.59-3.47 | 0.462 | 0.068 | -0.68-2.01 | 0.331 |
| Valvular disease | 0.064 | -0.55-2.12 | 0.247 | -0.004 | -1.78-1.71 | 0.968 | 0.063 | -1.13-3.01 | 0.372 |
| Supraventricular arrhythmias (SA+AFL) | -0.058 | -1.48-0.45 | 0.294 | -0.086 | -2.46-0.85 | 0.336 | -0.030 | -1.39-0.90 | 0.671 |
| Ventricular arrhythmias | 0.095 | -0.15-2.30 | 0.084 | 0.097 | -1.01-3.47 | 0.279 | 0.122 | -0.12-2.63 | 0.083 |
| *CIEDs* | 0.092 | -0.20-2.46 | 0.095 | 0.144 | -0.40-4.06 | 0.107 | 0.070 | -0.79-2.39 | 0.319 |
| Anti-bradycardia pacemaker | 0.011 | -1.36-1.67 | 0.836 | 0.034 | -2.04-3.02 | 0.704 | 0.001 | -1.80-1.82 | 0.991 |
| ICD | 0.121 | 0.56-9.59 | 0.028 | 0.274 | 3.83-16.40 | 0.002 | -0.007 | -6.43-5.78 | 0.917 |
| CRT | 0.112 | 0.13-6.54 | 0.041 | 0.055 | -4.48-8.57 | 0.536 | 0.159 | 0.57-7.57 | 0.023 |
| Peripheral artery disease | 0.206 | 2.96-9.27 | <0.001 | 0.079 | -2.56-6.69 | 0.379 | 0.313 | 5.66-13.88 | <0.001 |
| Diabetes mellitus type II | 0.120 | 0.10-1.87 | 0.029 | 0.111 | -0.52-2.29 | 0.213 | 0.116 | -0.17-2.03 | 0.099 |
| Prior stroke/TIA | 0.008 | -1.69-1.94 | 0.890 | 0.087 | -1.28-3.76 | 0.331 | -0.094 | -4.22-0.81 | 0.182 |
| Chronic kidney disease | 0.135 | 0.32-2.83 | 0.014 | 0.052 | -1.67-3.08 | 0.560 | 0.212 | 0.79-3.59 | 0.002 |
| COPD | 0.2016 | 1.37-4.03 | <0.001 | 0.116 | -0.77-3.71 | 0.196 | 0.294 | 1.90-5.03 | <0.001 |
| Malignancy | 0.147 | -0.13-1.59 | 0.008 | 0.087 | -1.28-3.76 | 0.331 | 0.190 | 0.77-4.65 | 0.006 |
| Thyroid dysfunction | 0.091 | -0.13-1.59 | 0.097 | 0.088 | -0.66-1.97 | 0.324 | 0.058 | -0.64-1.57 | 0.410 |
| Hyperlipoproteinemia | 1.33 | 0.18-1.65 | 0.015 | 0.160 | -0.10-2.31 | 0.073 | 0.127 | -0.07-1.71 | 0.070 |
| Other conditions | 0.116 | 0.08-2.28 | 0.035 | 0.035 | -1.53-2.28 | 0.695 | 0.184 | 0.45-3.01 | 0.008 |
| CHA_2_DS_2_-VASc score | 0.319 | 0.47-0.92 | <0.001 | 0.188 | 0.04-0.95 | 0.035 | 0.286 | 0.32-0.89 | <0.001 |
| >1 non-sex CHA_2_DS_2_-VASc risk factors | 0.162 | 0.61-2.99 | 0.003 | 0.146 | -0.45-4.93 | 0.102 | 0.137 | -0.01-2.57 | 0.051 |
| *Current medication* | | | | | | | | | |
| OAC | 0.118 | 0.12-2.48 | 0.032 | 0.128 | -0.50-3.16 | 0.153 | 0.134 | -0.04-2.94 | 0.056 |
| VKA | 0.159 | 0.34-1.75 | 0.004 | 0.098 | -0.51-1.80 | 0.273 | 0.222 | 0.54-2.23 | 0.001 |
| NOAC | -0.093 | -1.39-0.10 | 0.092 | -0.019 | -1.35-1.09 | 0.836 | -0.152 | -1.89-(-0.10) | 0.030 |
| OAC treatment duration (years) | 0.035 | -0.07-0.13 | 0.546 | 0.055 | -0.14-0.25 | 0.570 | 0.056 | -0.07-0.16 | 0.452 |
| ASA | 0.026 | -0.87-1.42 | 0.638 | 0.080 | -1.00-2.68 | 0.369 | -0.015 | -1.55-1.26 | 0.835 |
| P2Y_12_ inhibitor | 0.106 | -0.02-2.54 | 0.054 | 0.142 | -0.45-4.26 | 0.112 | 0.114 | -0.25-2.67 | 0.103 |
| Beta blocker | -0.097 | -1.66-0.09 | 0.077 | -0.023 | -1.57-1.22 | 0.801 | -0.139 | -2.16-(-0.02) | 0.047 |
| Non-DHP Ca blocker | 0.160 | 1.11-5.64 | 0.004 | 0.272 | 2.18-9.50 | 0.002 | 0.097 | -0.82-4.67 | 0.169 |
| Digitalis | 0.049 | -0.96-2.54 | 0.373 | -0.010 | -3.15-2.79 | 0.907 | 0.094 | -0.66-3.47 | 0.182 |
| Antiarrhythmic drugs | 0.020 | -0.58-0.85 | 0.71 | 0.092 | -0.57-1.80 | 0.306 | -0.041 | -1.12-0.60 | 0.556 |
| Mexiletine | ---- | ---- | ---- | ---- | ---- | ---- | ---- | ---- | ---- |
| Propafenone | -0.082 | -1.98-0.28 | 0.138 | -0.146 | --3.25-0.29 | 0.101 | -0.051 | -1.92-0.88 | 0.465 |
| Flecainide | -0.050 | -1.88-0.69 | 0.362 | 0.012 | -2.21-2.54 | 0.890 | -0.067 | -2.17-0.76 | 0.344 |
| Sotalol | -0.038 | -5.01-2.42 | 0.492 | -0.076 | -6.63-2.63 | 0.394 | -0.030 | -7.44-4.78 | 0.668 |
| Amiodarone | 0.108 | 0.004-1.44 | 0.049 | 0.199 | 0.17-2.47 | 0.025 | 0.034 | -0.66-1.10 | 0.626 |
| DHP Ca blocker | 0.069 | -0.27-1.22 | 0.210 | 0.082 | -0.63-1.70 | 0.362 | -0.002 | -0.96-0.94 | 0.982 |
| ACEI/ARB | 0.105 | -0.02-1.51 | 0.057 | 0.155 | -0.14-2.31 | 0.083 | 0.088 | -0.34-1.54 | 0.210 |
| Diuretic | 0.166 | 0.39-1.79 | 0.002 | 0.076 | -0.67-1.69 | 0.395 | 0.208 | 0.45-2.12 | 0.003 |
| Spironolactone | 0.179 | 0.55-2.16 | 0.001 | 0.211 | 0.31-3.15 | 0.017 | 0.207 | 0.49-2.36 | 0.003 |
| Statins | 0.055 | -0.35-1.08 | 0.318 | -0.025 | -1.33-1.00 | 0.778 | 0.085 | -0.34-1.41 | 0.229 |
| Sedative | 0.150 | 0.45-2.72 | 0.006 | 0.003 | -1.87-1.94 | 0.971 | 0.256 | 1.21-1.87 | <0.001 |
| PPI | 0.118 | 0.07-1.62 | 0.032 | 0.111 | -0.49-2.16 | 0.213 | 0.154 | 0.11-1.94 | 0.028 |
| Insulin | 0.123 | 0.25-3.85 | 0.026 | 0.092 | -1.13-3.60 | 0.304 | 0.117 | -0.41-507 | 0.095 |
| Oral antidiabetic drug | 0.086 | -0.20-1.76 | 0.117 | 0.087 | -0.77-2.27 | 0.329 | 0.069 | -0.62-1.86 | 0.325 |
| Other medications | 0.306 | 1.35-2.73 | <0.001 | 0.247 | 0.50-2.76 | 0.005 | 0.329 | 1.28-2.96 | <0.001 |
| *Non-pharmacological treatment* | | | | | | | | | |
| Ablation/ECV | -0.143 | -1.66-(-0.24) | 0.009 | 0.022 | -1.10-1.41 | 0.805 | -0.186 | -1.99-(-0.31) | 0.008 |
| ECV AF | -0.174 | -2.01-(-0.48) | 0.002 | -0.057 | -1.95-1.00 | 0.525 | -0.181 | -2.04-(-0.29) | 0.010 |
| ECV AFL | 0.055 | -1.42-4.35 | 0.318 | 0.156 | -0.50-8.68 | 0.079 | -0.013 | -3.87-3.22 | 0.855 |
| AF Ablation | -0.094 | -1.76-0.12 | 0.089 | -0.038 | -2.01-1.30 | 0.675 | -0.112 | -1.99-0.21 | 0.112 |
| AFL Ablation | -0.055 | -3.26-1.07 | 0.320 | ---- | ---- | ---- | -0.030 | -2.53-1.62 | 0.666 |
| Ablation other arrhythmias | -0.047 | -6.51-2.57 | 0.395 | -0.081 | -9.51-3.52 | 0.365 | -0.300 | -7.44-4.78 | 0.668 |
| *Multimorbidity and polypharmacy* | | | | | | | | | |
| Polypharmacy | 0.235 | 0.93-2.45 | <0.001 | 0.259 | 0.67-3.26 | 0.003 | 0.205 | 0.46-2.27 | 0.003 |
| N of drugs | 0.345 | 0.29-0.53 | <0.001 | 0.328 | 0.20-0.61 | <0.001 | 0.352 | 0.25-0.53 | <0.001 |
| N of pills | 0.331 | 0.23-0.43 | <0.001 | 0.259 | 0.10-0.46 | 0.003 | 0.377 | 0.23-0.46 | <0.001 |
| N of drugs without OAC | 0.338 | 0.29-0.54 | <0.001 | 0.317 | 0.19-0.62 | <0.001 | 0347 | 0.25-0.55 | <0.001 |
| N of pills without OAC | 0.327 | 0.23-0.43 | <0.001 | 0.248 | 0.08-0.46 | 0.005 | 0.374 | 0.23-0.47 | <0.001 |
| Parenteral drug use | 0.112 | 0.06-3.32 | 0.043 | 0.092 | -1.02-3.26 | 0.301 | 0.085 | -0.96-4.07 | 0.224 |
| N of parenteral applications daily | 0.084 | -0.13-1.07 | 0.127 | 0.092 | -0.36-1.16 | 0.304 | 0.024 | -0.81-1.14 | 0.738 |
| N of comorbidities | 0.284 | 0.33-0.72 | <0.001 | 0.169 | -0.01-0.63 | 0.058 | 0.371 | 0.43-0.88 | <0.001 |
| Patients with multimorbidity (without SA/ VA) | 0.122 | 0.20-3.06 | 0.026 | 0.085 | -1.53-4.38 | 0.343 | 0.120 | -0.21-2.94 | 0.088 |
| ***Treatment burden*** | | | | | | | | | |
| Total TB | 0.267 | 0.02-0.05 | <0.001 | 0.271 | 0.01-0.06 | 0.002 | 0.190 | 0.01-0.05 | 0.006 |
| TB ≥59 | 0.220 | 0.83-2.38 | <0.001 | 0.197 | 0.16-2.49 | 0.027 | 0.171 | 0.26-2.34 | 0.014 |
| TB ≤26 | -0.132 | -1.87-(-0.19) | 0.017 | -0.048 | -1.97-1.13 | 0.595 | -0.146 | -2.00-(-0.06) | 0.038 |
| ***Questions about OAC-related treatment burden*** | | | | | | | | | |
| Q1 | 0.013 | -0.24-0.31 | 0.808 | 0.047 | -0.28-0.47 | 0.599 | -0054 | -0.53-0.23 | 0.439 |
| Q2 | 0.039 | -0.11-0.24 | 0.480 | 0.083 | -0.16-0.44 | 0.353 | 0.022 | -0.18-0.24 | 0.755 |
| Q3 | 0.007 | -0.17-0.19 | 0.893 | 0.063 | -0.18-0.38 | 0.481 | -0.052 | -0.30-0.14 | 0.460 |
| Q4 | 0.054 | -0.09-0.28 | 0.326 | 0.107 | -0.11-0.45 | 0.232 | -0.002 | -0.24-0.23 | 0.977 |
| ***Questions about other drugs-related treatment burden*** | | | | | | | | | |
| Q1 | 0.107 | -0.01-033 | 0.052 | -0.017 | -0.27-0.22 | 0.851 | 0.177 | 0.06-0.48 | 0.011 |
| Q2 | 0.149 | 0.06-0.35 | 0.007 | 0.158 | -0.02-0.40 | 0.075 | 0.088 | -0.08-0.34 | 0.211 |
| Q3 | 0.100 | -0.01-0.28 | 0.069 | 0.150 | -0.03-0.41 | 0.093 | 0.027 | -0.16-0.23 | 0.701 |
| Q4 | 0.094 | -0.02-0.27 | 0.088 | 0.142 | -0.04-0.38 | 0.110 | 0.06 | -0.19-0.21 | 0.928 |
| ***Questions about other aspects of treatment burden*** | | | | | | | | | |
| Q1 | 0.226 | 0.13-0.36 | <0.001 | 0.201 | 0.03-0.37 | 0.024 | 0.214 | 0.09-0.38 | 0.002 |
| Q2 | 0.147 | 0.04-0.28 | 0.007 | 0.135 | -0.04-0.30 | 0.131 | 0.108 | -0.03-0.28 | 0.125 |
| Q3 | 0.232 | 0.12-0.33 | <0.001 | 0.248 | 0.07-0.40 | 0.005 | 0.167 | 0.03-0.29 | 0.017 |
| Q4 | 0.038 | -0.06-0.12 | 0.495 | 0.059 | -0.10-0.20 | 0.510 | -0.006 | -0.12-0.11 | 0.937 |
| Q5 | 0.040 | -0.06-0.13 | 0.467 | 0.049 | -0.11-0.19 | 0.587 | -0.010 | -0.13-0.11 | 0.892 |
| Q6 | 0.146 | 0.04-0.26 | 0.008 | 0.126 | -0.05-0.29 | 0.159 | 0.058 | -0.09-0.22 | 0.409 |
| Q7 | 0.423 | 0.35-0.56 | <0.001 | 0.411 | 0.25-0.56 | <0.001 | 0.391 | 0.29-0.58 | <0.001 |
| Q8 | 0.309 | 0.32-0.65 | <0.001 | 0.266 | 0.12-0.55 | 0.002 | 0.307 | 0.34-0.85 | <0.001 |
| Q9 | 0.241 | 0.14-0.37 | <0.001 | 0.154 | -0.02-0.31 | 0.084 | 0.245 | 0.13-0.43 | <0.001 |

**AF:** Atrial fibrillation; **LVEF:** Left ventricular ejection fraction; **ACS:** Acute coronary syndrome; **MI:** Myocardial infarction; **CAD:** Coronary artery disease; **PCI:** Percutaneous coronary intervention; **CABG:** Coronary artery bypass grafting; **AFL:** Atrial flutter; **CIED:** Cardiac implantable electronic devices; **ICD:** Implantable cardioverter defibrillator; **CRT:** Cardiac resynchronisation therapy; **TIA:** Transient ischemic attack, **COPD:** Chronic obstructive pulmonary disease; **OAC:** Oral anticoagulant therapy; **VKA:** Vitamin K antagonist; **NOAC:** Non-vitamin K antagonist oral anticoagulant; **ASA:** Acetylsalicylic acid; **DHP:** Dihydropyridine; **ACEI:** Angiotensin converting enzyme inhibitor; **ARB:** Angiotensin receptor inhibitor; **PPI:** Proton pump inhibitor; **ECV:** electrical cardioversion; **SA:** Supraventricular arrhythmias; **VA:** Ventricular arrhythmias; **N:** Number; **Q:** Question.

**Table S7. AF of highest quartile QOL ≥6. In AF**

| **Variable** | **AF patients**  **n=59 (%)** | | | **Female**  **n=36 (61.0)** | | | **Male**  **n=23 (39.0)** | | |
| --- | --- | --- | --- | --- | --- | --- | --- | --- | --- |
|  | **OR** | **CI 95%** | **P value** | **OR** | **CI 95%** | **P value** | **OR** | **CI 95%** | **P value** |
| Age | 1.067 | 1.03-1.10 | <0.001 | 1.050 | 0.999-1.10 | 0.053 | 1.070 | 1.02-1.12 | 0.004 |
| Age ≤40 | 0.764 | 0.09-6.447 | 0.805 | 0.000 | 0.00---- | 1.000 | 1.600 | 0.18-14.33 | 0.674 |
| Age ≤45 | 0.318 | 0.04-2.47 | 0.273 | 0.000 | 0.00---- | 0.999 | 0.702 | 0.09-5.71 | 0.741 |
| Age ≤47 | 0.243 | 0.03-1.86 | 0.173 | 0.000 | 0.00---- | 0.999 | 0.542 | 0.07-4.33 | 0.564 |
| Age ≤48 | 0.187 | 0.03-1.41 | 0.104 | 0.000 | 0.00---- | 0.999 | 0.388 | 0.05-3.04 | 0.367 |
| Age ≤49 | 0.170 | 0.02-1.28 | 0.086 | 0.000 | 0.00--- | 0.999 | 0.346 | 0.04-2.70 | 0.312 |
| Age ≤50 | 0.144 | 0.02-1.08 | 0.060 | 0.000 | 0.00---- | 0.999 | 0.297 | 0.04-2.31 | 0.246 |
| Age ≤64 | 0.358 | 0.19-0.69 | 0.002 | 0.643 | 0.26-1.59 | 0.338 | 0.269 | 0.10-0.76 | 0.013 |
| Age 65-74 | 1.169 | 0.66-2.06 | 0.588 | 0.875 | 0.40-1.90 | 0.735 | 1.278 | 0.53-3.07 | 0.583 |
| Age ≥75 | 2.925 | 1.54-5.57 | 0.001 | 1.915 | 0.79-4.63 | 0.149 | 4.063 | 1.54-10.73 | 0.005 |
| Age ≥80 | 2.880 | 1.21-6.88 | 0.017 | 1.288 | 0.30-5.45 | 0.731 | 6.035 | 1.95-18.65 | 0.002 |
| *Education degree* | | | | | | | | | |
| Elementary | 2.857 | 1.42-5.76 | 0.003 | 2.640 | 1.05-6.63 | 0.039 | 2.171 | 0.66-7.17 | 0.203 |
| High school | 0.754 | 0.43-1.33 | 0.328 | 0.640 | 0.29-1.40 | 0.262 | 0.907 | 0.38-2.16 | 0.825 |
| College | 0.781 | 0.33-1.84 | 0.572 | 0.968 | 0.32-2.94 | 0.954 | 0.543 | 0.12-2.45 | 0.427 |
| University | 0.745 | 0.37-1.52 | 0.418 | 0.702 | 0.24-2.07 | 0.522 | 1.006 | 0.38-2.70 | 0.990 |
| *Employment status* | | | | | | | | | |
| Employed  Unemployed  Retired | 0.347 | 0.15-0.80 | 0.013 | 1.012 | 0.30-3.46 | 0.984 | 0.261 | 0.08-0.91 | 0.035 |
|  | 0.675 | 0.19-2.35 | 0.537 | 0.610 | 0.12-3.02 | 0.545 | 0.640 | 0.08-5.16 | 0.675 |
|  | 2.673 | 1.30-5.51 | 0.008 | 1.233 | 0.45-3.41 | 0.687 | 3.597 | 1.18-11.00 | 0.025 |
| *Marital status* | | | | | | | | | |
| Married/living with a partner | 0.424 | 0.23-0.77 | 0.005 | 0.358 | 0.16-0.80 | 0.012 | 0.833 | 0.29-2.40 | 0.735 |
| Alone/divorced | 0.449 | 0.13-1.53 | 0.200 | 0.706 | 0.14-357 | 0.674 | 0.329 | 0.04-2.56 | 0.288 |
| Widow(er) | 4.297 | 2.22-8.33 | <0.001 | 3.482 | 1.50-8.09 | 0.004 | 2.965 | 0.87-10.11 | 0.083 |
| *Cigarette smoking* | | | | | | | | | |
| Smoker  Former smoker  Non-smoker | 1.044 | 0.48-2.29 | 0.914 | 1.012 | 0.32-3.46 | 0.984 | 1.398 | 0.48-4.06 | 0.538 |
|  | 1.271 | 0.69-2.33 | 0.439 | 1.013 | 0.36-2.86 | 0.980 | 2.688 | 1.11-6.49 | 0.028 |
|  | 0.799 | 0.45-1.41 | 0.436 | 0.985 | 0.42-2.33 | 0.972 | 0.269 | 0.10-0.76 | 0.013 |
| *Functional mobility* | | | | | | | | | |
| Fully mobile  Mobile with help  Immobile | 0.048 | 0.02-0.15 | <0.001 | 0.039 | 0.01-0.33 | 0.003 | 0.048 | 0.01-0.21 | <0.001 |
|  | 20.844 | 6.57-66.17 | <0.001 | 25.714 | 3.08-214.59 | 0.003 | 20.941 | 4.80-91.31 | <0.001 |
|  | ----- | ----- | ----- | ----- | ----- | ----- | ----- | ----- | ----- |
| *AF characteristics* | | | | | | | | | |
| Total AF history (years) | 1.018 | 0.98-1.06 | 0.391 | 0.997 | 0.93-1.07 | 0.930 | 1.046 | 0.996-1.10 | 0.073 |
| Permanent AF | 1.181 | 0.64-2.17 | 0.590 | 1.320 | 0.57-3.03 | 0.513 | 1.625 | 1.27-2.08 | <0.001 |
| *Comorbid conditions* | | | | | | | | | |
| Hypertension | 2.188 | 0.89-5.36 | 0.086 | 3.627 | 0.79-16.66 | 0.098 | 1.262 | 0.41-3.93 | 0.688 |
| Heart failure | 3.920 | 1.85-8.32 | <0.001 | 0.706 | 0.14-3.57 | 0.674 | 14.098 | 5.22-38.09 | <0.001 |
| LVEF <50% | 1.369 | 0.66-2.86 | 0.404 | 0.491 | 0.06-4.36 | 0.524 | 3.326 | 1.35-8.22 | 0.009 |
| *Ischemic heart disease* | 1.097 | 0.53-2.27 | 0.803 | 0.598 | 0.16-2.26 | 0.449 | 2.225 | 0.87-5.66 | 0.093 |
| Recent ACS | ---- | ----- | 0.999 | ---- | ----- | 1.000 | ---- | ----- | 1.000 |
| Prior MI | 0.719 | 0.24-2.15 | 0.554 | 0.000 | 0.00---- | 0.999 | 1.522 | 0.47-4.89 | 0.481 |
| Chronic stable CAD | 1.067 | 0.29-3.87 | 0.921 | 1.012 | 0.19-5.47 | 0.989 | 0.983 | 0.12-8.24 | 0.987 |
| PCI/Balloon angioplasty | 1.457 | 0.59-3.58 | 0.411 | 0.491 | 0.06-4.36 | 0.524 | 3.196 | 1.12-914 | 0.030 |
| CABG | 1.556 | 0.31-7.91 | 0.594 | ---- | ---- | ---- | 2.778 | 0.53-14.65 | 0.229 |
| Cardiomyopathy | 1.170 | 0.46-3.00 | 0.744 | 1.977 | 0.42-9.31 | 0.389 | 1.207 | 0.33-4.43 | 0.776 |
| Valvular disease | 1.507 | 0.58-3.95 | 0.404 | 0.823 | 0.25-2.74 | 0.751 | 2.367 | 0.46-12.15 | 0.302 |
| Supraventricular arrhythmias (SA+AFL) | 0.339 | 0.12-0.98 | 0.046 | 0.461 | 0.13-1.70 | 0.244 | 0.204 | 0.03-1.57 | 0.126 |
| Ventricular arrhythmias | 1.783 | 0.75-4.23 | 0.189 | 2.150 | 0.54-8.51 | 0.276 | 2.031 | 0.62-6.66 | 0.243 |
| *CIEDs* | 2.353 | 0.96-5.74 | 0.060 | 3.508 | 0.89-13.91 | 0.074 | 1.938 | 0.51-7.39 | 0.232 |
| Anti-bradycardia pacemaker | 1.246 | 0.40-3.90 | 0.705 | 1.977 | 0.42-9.31 | 0.389 | 0.702 | 0.09-5.71 | 0.741 |
| ICD | 4.672 | 0.29-75.79 | 0.278 | ---- | ---- | 1.000 | 0.000 | 0.00--- | 1.000 |
| CRT | 14.518 | 1.48-142.13 | 0.022 | ---- | ---- | 1.000 | 17.143 | 1.49-197.20 | 0.023 |
| Peripheral artery disease | 14.518 | 1.48-142.13 | 0.022 | 2.571 | 0.16-42.25 | 0.508 | ---- | ---- | 0.999 |
| Diabetes mellitus type II | 1.551 | 0.80-301 | 0.194 | 1.674 | 0.68-4.12 | 0.262 | 1.293 | 0.45-3.74 | 0.635 |
| Prior stroke/TIA | 1.404 | 0.37-5.27 | 0.615 | 1.977 | 0.42-9.31 | 0.389 | 0.000 | 0.00--- | 0.999 |
| Chronic kidney disease | 2.880 | 1.25-6.61 | 0.013 | 1.564 | 0.35-6.91 | 0.556 | 5.654 | 1.97-16.19 | 0.001 |
| COPD | 4.566 | 1.93-10.79 | 0.001 | 3.508 | 0.89-13.91 | 0.074 | 6.745 | 2.14-21.24 | 0.001 |
| Malignancy | 3.527 | 1.28-9.69 | 0.015 | 1.977 | 0.42-9.31 | 0.389 | 6.140 | 1.59-23.71 | 0.008 |
| Thyroid dysfunction | 1.345 | 0.70-2.59 | 0.376 | 1.137 | 0.48-2.71 | 0.772 | 1.293 | 0.45-3.74 | 0.635 |
| Hyperlipoproteinemia | 2.315 | 1.31-4.10 | 0.004 | 2.640 | 1.19-5.87 | 0.017 | 2.256 | 0.94-5.41 | 0.068 |
| Other conditions | 1.508 | 0.67-3.38 | 0.318 | 1.139 | 0.33-3.96 | 0.838 | 2.236 | 0.75-6.68 | 0.150 |
| CHA_2_DS_2_-VASc score | 1.615 | 1.32-1.98 | <0.001 | 1.332 | 0.97-1.83 | 0.075 | 1.656 | 1.22-2.25 | 0.001 |
| >1 non-sex CHA_2_DS_2_-VASc risk factors | ----- | ---- | 0.998 | ---- | ---- | 0.989 | ---- | ---- | 0.999 |
| *Current medication* | | | | | | | | | |
| OAC | 7.461 | 0.998-55.78 | 0.050 | 5.833 | 0.73-46.35 | 0.095 | ---- | ---- | 0.998 |
| VKA | 1.738 | 0.96-3.15 | 0.069 | 1.656 | 0.75-3.67 | 0.213 | 2.194 | 0.83-5.82 | 0.114 |
| NOAC | 0.859 | 0.47-1.58 | 0.624 | 0.968 | 0.43-2.19 | 0.937 | 0.694 | 0.26-1.85 | 0.466 |
| OAC treatment duration (years) | 1.002 | 0.93-1.58 | 0.961 | 0.996 | 0.88-1.12 | 0.954 | 1.028 | 0.93-1.85 | 0.466 |
| ASA | 0.949 | 0.38-2.40 | 0.911 | 1.470 | 0.46-4.73 | 0.519 | 0.366 | 0.05-2.86 | 0.338 |
| P2Y_12_ inhibitor | 1.353 | 0.52-3.51 | 0.535 | 2.719 | 0.64-11.52 | 0.175 | 0.919 | 0.20-4.26 | 0.914 |
| Beta blocker | 0.693 | 0.36-1.34 | 0.276 | 0.792 | 0.32-1.96 | 0.614 | 0.632 | 0.23-1.73 | 0.370 |
| Non-DHP Ca blocker | 4.873 | 1.18-20.08 | 0.028 | ---- | ---- | 0.994 | 2.011 | 0.22-12.15 | 0.302 |
| Digitalis | 1.905 | 0.58-6.30 | 0.290 | 1.725 | 0.28-10.78 | 0.560 | 2.367 | 0.46-12.15 | 0.302 |
| Antiarrhythmic drugs | 0.938 | 0.53-1.66 | 0.825 | 0.835 | 0.38-1.84 | 0.654 | 0.963 | 0.40-2.31 | 0.932 |
| Mexiletine | ---- | ---- | ---- | ---- | ---- | ---- | ---- | ---- | ---- |
| Propafenone | 0.388 | 0.12-1.31 | 0.127 | 0.353 | 0.08-1.65 | 0.186 | 0.366 | 0.05-2.86 | 0.338 |
| Flecainide | 0.554 | 0.16-1.90 | 0.348 | 1.564 | 0.35-6.91 | 0.556 | 0.000 | 0.00---- | 0.998 |
| Sotalol | 0.000 | 0.00--- | 0.999 | 0.000 | 0.00--- | 0.999 | 0.000 | 0.00--- | 0.999 |
| Amiodarone | 1.529 | 0.87-2.70 | 0.142 | 1.168 | 0.54-2.53 | 0.697 | 1.994 | 0.83-4.78 | 0.121 |
| DHP Ca blocker | 1.118 | 0.62-2.02 | 0.712 | 1.528 | 0.70-3.32 | 0.285 | 0.353 | 0.10-1.24 | 0.104 |
| ACEI/ARB | 2.087 | 1.03-4.21 | 0.040 | 2.357 | 0.93-5.97 | 0.071 | 2.073 | 0.67-6.38 | 0.203 |
| Diuretic | 2.607 | 1.39-4.91 | 0.003 | 1.434 | 0.64-3.22 | 0.382 | 6.593 | 1.89-22.97 | 0.003 |
| Spironolactone | 2.836 | 1.57-5.14 | 0.001 | 3.000 | 1.21-7.44 | 0.018 | 4.172 | 1.71-10.19 | 0.002 |
| Statins | 1.281 | 0.73-2.26 | 0.391 | 0.833 | 0.38-1.82 | 0.647 | 1.906 | 0.80-4.55 | 0.149 |
| Sedative | 2.764 | 1.29-5.94 | 0.009 | 1.139 | 0.33-3.96 | 0.838 | 6.362 | 2.30-17.59 | <0.001 |
| PPI | 1.347 | 0.74-2.46 | 0.331 | 1.206 | 0.50-2.89 | 0.674 | 1.858 | 0.77-4.50 | 0.170 |
| Insulin | 3.056 | 0.96-9.70 | 0.058 | 2.719 | 0.64-11.52 | 0.175 | 2.011 | 0.22-18.81 | 0.540 |
| Oral antidiabetic drug | 1.184 | 0.55-2.53 | 0.663 | 1.223 | 0.45-3.30 | 0.691 | 0.936 | 0.26-3.38 | 0.920 |
| Other medications | 3.401 | 1.90-6.11 | <0.001 | 1.708 | 0.79-3.72 | 0.177 | 8.467 | 2.99-23.97 | <0.001 |
| *Non-pharmacological treatment* | | | | | | | | | |
| Ablation/ECV | 0.573 | 0.31-1.05 | 0.071 | 0.990 | 0.43-2.29 | 0.981 | 0.442 | 0.17-1.13 | 0.087 |
| ECV AF | 0.441 | 0.21-0.91 | 0.027 | 0.811 | 0.29-2.24 | 0.687 | 0.350 | 0.11-1.07 | 0.066 |
| ECV AFL | 3.146 | 0.51-19.26 | 0.215 | ---- | ---- | 0.999 | 0.000 | 0.00---- | 0.999 |
| AF Ablation | 0.411 | 0.16-1.08 | 0.071 | 0.461 | 0.13-1.70 | 0.244 | 0.397 | 0.09-1.77 | 0.227 |
| AFL Ablation | 0.569 | 0.07-4.64 | 0.598 | ---- | ----- | ---- | 0.983 | 0.12-8.24 | 0.987 |
| Ablation other arrhythmias | 0.000 | 0.00---- | 0.999 | 0.000 | 0.00---- | 0.999 | 0.000 | 0.00---- | 0.999 |
| *Multimorbidity and polypharmacy* | | | | | | | | | |
| Polypharmacy | 6.799 | 2.39-19.35 | <0.001 | 4.889 | 1.38-17.29 | 0.014 | 11.462 | 1.51-87.05 | 0.018 |
| N of drugs | 1.273 | 1.15-1.41 | <0.001 | 1.232 | 1.06-1.43 | 0.006 | 1.332 | 1.15-1.55 | <0.001 |
| N of pills | 1.201 | 1.10-1.31 | <0.001 | 1.139 | 1.00-1.29 | 0.045 | 1.278 | 1.13-1.45 | <0.001 |
| N of drugs without OAC | 1.274 | 1.15-1.42 | <0.001 | 1.219 | 1.05-1.42 | 0.012 | 1.347 | 1.15-1.58 | <0.001 |
| N of pills without OAC | 1.196 | 1.10-1.30 | <0.001 | 1.127 | 0.99-1.28 | 0.066 | 1278 | 1.13-1.45 | <0.001 |
| Parenteral drug use | 2.966 | 1.03-8.51 | 0.043 | 2.774 | 0.75-10.24 | 0.126 | 1.600 | 0.18-14.33 | 0.674 |
| N of parenteral applications daily | 1.431 | 0.98-2.10 | 0.067 | 1.482 | 0.92-2.38 | 0.105 | 0.832 | 0.24-2.90 | 0.773 |
| N of comorbidities | 1.330 | 1.14-1.55 | <0.001 | 1.163 | 0.94-1.44 | 0.165 | 1.625 | 1.27-2.08 | <0.001 |
| Patients with multimorbidity (without SA/ VA) | ---- | ---- | 0.998 | ---- | ---- | 0.999 | ---- | ---- | 0.998 |
| ***Treatment burden*** | | | | | | | | | |
| Total TB | 1.020 | 1.01-1.03 | <0.001 | 1.017 | 1.00-1.03 | 0.023 | 1.016 | 1.00-1.03 | 0.054 |
| TB ≥59 | 2.561 | 1.43-4.60 | 0.002 | 2.033 | 0.93-4.46 | 0.077 | 2.306 | 0.91-5.88 | 0.080 |
| TB ≤26 | 0.420 | 0.18-0..97 | 0.042 | 0.544 | 0.17-1.75 | 0.306 | 0.404 | 0.12-1.42 | 0.158 |
| ***Questions about OAC-related treatment burden*** | | | | | | | | | |
| Q1 | 0.953 | 0.75-1.21 | 0.688 | 0.993 | 0.77-1.28 | 0.957 | 0.529 | 0.15-1.83 | 0.315 |
| Q2 | 1.009 | 0.88-1.16 | 0.895 | 1.038 | 0.86-1.26 | 0.709 | 0.992 | 0.80-1.23 | 0.938 |
| Q3 | 0.998 | 0.87-1.15 | 0.980 | 1.075 | 0.90-1.28 | 0.424 | 0.764 | 0.49-1.19 | 0.233 |
| Q4 | 1.081 | 0.95-1.23 | 0.248 | 1.137 | 0.96-1.35 | 0.145 | 0.961 | 0.74-1.25 | 0.764 |
| ***Questions about other drugs-related treatment burden*** | | | | | | | | | |
| Q1 | 1.041 | 0.92-1.18 | 0.528 | 0.931 | 0.78-1.12 | 0.441 | 1.150 | 0.96-1.37 | 0.121 |
| Q2 | 1.114 | 1.00-1.24 | 0.045 | 1.064 | 0.93-1.22 | 0.366 | 1.121 | 0.94-1.34 | 0.205 |
| Q3 | 1.113 | 1.00-1.24 | 0.045 | 1.121 | 0.97-1.29 | 0.112 | 1.060 | 0.89-1.26 | 0.516 |
| Q4 | 1.087 | 0.98-1.21 | 0.121 | 1.065 | 0.93-1.22 | 0.362 | 1.059 | 0.88-1.27 | 0.541 |
| ***Questions about other aspects of treatment burden*** | | | | | | | | | |
| Q1 | 1.182 | 1.09-1.29 | <0.001 | 1.108 | 0.99-1.24 | 0.078 | 1.255 | 1.10-1.43 | 0.001 |
| Q2 | 1.151 | 1.06-1.25 | 0.001 | 1.117 | 0.999-1.25 | 0.051 | 1.136 | 1.00-1.28 | 0.045 |
| Q3 | 1.189 | 1.10-1.29 | <0.001 | 1.200 | 1.07-1.35 | 0.002 | 1.136 | 1.00-1.29 | 0.045 |
| Q4 | 1.028 | 0.96-1.11 | 0.462 | 1.030 | 0.93-1.14 | 0.563 | 1.002 | 0.90-1.12 | 0.973 |
| Q5 | 0.981 | 0.91-1.06 | 0.620 | 0.993 | 0.90-1.10 | 0.882 | 0.919 | 0.81-1.05 | 0.205 |
| Q6 | 1.079 | 0.99-1.18 | 0.077 | 1.043 | 0.93-1.17 | 0.463 | 1.025 | 0.88-1.19 | 0.752 |
| Q7 | 1.253 | 1.15-1.37 | <0.001 | 1.177 | 1.05-1.32 | 0.005 | 1.303 | 1.14-1.49 | <0.001 |
| Q8 | 1.268 | 1.13-1.42 | <0.001 | 1.187 | 1.03-1.37 | 0.016 | 1.314 | 1.09-1.59 | 0.005 |
| Q9 | 1.143 | 1.05-1.24 | 0.002 | 1.100 | 0.98-1.23 | 0.093 | 1.133 | 0.99-1.30 | 0.078 |

**AF:** Atrial fibrillation; **LVEF:** Left ventricular ejection fraction; **ACS:** Acute coronary syndrome; **MI:** Myocardial infarction; **CAD:** Coronary artery disease; **PCI:** Percutaneous coronary intervention; **CABG:** Coronary artery bypass grafting; **AFL:** Atrial flutter; **CIED:** Cardiac implantable electronic devices; **ICD:** Implantable cardioverter defibrillator; **CRT:** Cardiac resynchronisation therapy; **TIA:** Transient ischemic attack, **COPD:** Chronic obstructive pulmonary disease; **OAC:** Oral anticoagulant therapy; **VKA:** Vitamin K antagonist; **NOAC:** Non-vitamin K antagonist oral anticoagulant; **ASA:** Acetylsalicylic acid; **DHP:** Dihydropyridine; **ACEI:** Angiotensin converting enzyme inhibitor; **ARB:** Angiotensin receptor inhibitor; **PPI:** Proton pump inhibitor; **ECV:** electrical cardioversion; **SA:** Supraventricular arrhythmias; **VA:** Ventricular arrhythmias; **N:** Number; **Q:** Question.

**Table S8. AF QOL ≤1 in AF**

| **Variable** | **AF patients**  **n=150 (%)** | | | **Female**  **n=36 (24.0)** | | | **Male**  **n=114 (76.0)** | | |
| --- | --- | --- | --- | --- | --- | --- | --- | --- | --- |
|  | **OR** | **CI 95%** | **P value** | **OR** | **CI 95%** | **P value** | **OR** | **CI 95%** | **P value** |
| Age | 0.953 | 0.93-0.98 | <0.001 | 0.960 | 0.92-1.00 | 0.068 | 0.960 | 0.93-0.99 | 0.004 |
| Age ≤40 | 1.626 | 0.36-7.38 | 0.529 | ---- | ----- | 1.000 | 0.784 | 0.15-3.98 | 0.769 |
| Age ≤45 | 1.400 | 0.50-3.96 | 0.525 | 5.29 | 0.47-60.30 | 0.179 | 0.778 | 0.24-2.50 | 0.673 |
| Age ≤47 | 1.711 | 0.67-4.37 | 0.261 | 8.182 | 0.82-81.45 | 0.073 | 0.895 | 0.31-2.57 | 0.836 |
| Age ≤48 | 2.582 | 1.07-6.21 | 0.034 | 8.182 | 0.82-81.45 | 0.073 | 1.526 | 0.58-4.00 | 0.390 |
| Age ≤49 | 2.949 | 1.24-6.99 | 0.014 | 8.182 | 0.82-81.45 | 0.073 | 1.797 | 0.70-4.62 | 0.224 |
| Age ≤50 | 2.631 | 1.19-5.81 | 0.017 | 4.045 | 0.65-25.30 | 0.135 | 1.796 | 074-4.38 | 0.197 |
| Age ≤64 | 2.121 | 1.36-3.32 | 0.001 | 0.962 | 0.41-2.27 | 0.329 | 2.404 | 1.36-4.25 | 0.003 |
| Age 65-74 | 0.823 | 0.53-1.28 | 0.384 | 1.916 | 0.87-4.21 | 0.105 | 0.625 | 0.35-1.11 | 0.106 |
| Age ≥75 | 0.368 | 0.20-0.69 | 0.002 | 0.349 | 0.11-1.09 | 0.070 | 0.427 | 0.19-0.96 | 0.039 |
| Age ≥80 | 0.278 | 0.10-0.76 | 0.012 | 0.706 | 0.14-3.57 | 0.674 | 0.160 | 0.04-0.58 | 0.005 |
| *Education degree* | | | | | | | | | |
| Elementary | 0.582 | 0.30-1.13 | 0.111 | 0.811 | 0.29-2.24 | 0.687 | 0.616 | 0.24-1.56 | 0.306 |
| High school | 1.115 | 0.72-172 | 0.623 | 1.194 | 0.55-2.59 | 0.653 | 1.083 | 0.62-1.88 | 0.778 |
| College | 0.877 | 0.47-1.64 | 0.681 | 0.688 | 0.21-2.25 | 0.535 | 0.967 | 0.44-2.13 | 0.934 |
| University | 1.320 | 0.79-2.21 | 0.291 | 1.223 | 0.45-3.30 | 0.691 | 1.154 | 0.61-2.18 | 0.657 |
| *Employment status* | | | | | | | | | |
| Employed  Unemployed  Retired | 2.948 | 1.76-4.95 | <0.001 | 1.470 | 0.46-4.73 | 0.519 | 2.638 | 1.42-4.91 | 0.002 |
|  | 0.923 | 0.39-2.17 | 0.854 | 1.091 | 0.27-4.47 | 0.904 | 0.916 | 0.30-2.83 | 0.879 |
|  | 0.406 | 0.25-0.65 | <0.001 | 0.747 | 0.29-1.94 | 0.548 | 0.421 | 0.23-0.76 | 0.004 |
| *Marital status* | | | | | | | | | |
| Married/living with a partner | 1.682 | 1.00-2.82 | 0.049 | 0.984 | 0.43-2.23 | 0.968 | 1.846 | 0.91-3.74 | 0.088 |
| Alone/divorced | 1.231 | 0.59-2.55 | 0.576 | 0.706 | 0.14-3.57 | 0.674 | 1.260 | 0.52-3.06 | 0.610 |
| Widow(er) | 0.382 | 0.19-0.75 | 0.005 | 1.137 | 0.48-2.71 | 0.772 | 0.160 | 0.04-0.58 | 0.005 |
| *Cigarette smoking* | | | | | | | | | |
| Smoker  Former smoker  Non-smoker | 0.730 | 0.39-1.36 | 0.320 | 1.012 | 0.30-3.46 | 0.984 | 0.530 | 0.25-1.11 | 0.091 |
|  | 0.826 | 0.51-1.34 | 0.440 | 0.223 | 0.05-1.01 | 0.052 | 0.755 | 0.42-1.35 | 0.340 |
|  | 1.374 | 0.89-2.13 | 0.157 | 2.339 | 0.88-6.24 | 0.090 | 1.874 | 1.07-3.29 | 0.028 |
| *Functional mobility* | | | | | | | | | |
| Fully mobile  Mobile with help  Immobile | 14.45 | 2.03-117.48 | 0.008 | ----- | ---- | 0.999 | 11.024 | 1.35-89.87 | 0.025 |
|  | 0.065 | 0.01-0.49 | 0.008 | ---- | ---- | 0.998 | 0.091 | 0.01-0.74 | 0.025 |
|  | ----- | ----- | ----- | ----- | ----- | ----- | ----- | ----- | ----- |
| *AF characteristics* | | | | | | | | | |
| Total AF history (years) | 1.007 | 0.97-1.04 | 0.688 | 1.035 | 0.97-1.11 | 0.312 | 0.987 | 0.95-1.03 | 0.515 |
| Permanent AF | 0.945 | 0.59-1.52 | 0.816 | 1.881 | 0.83-4.27 | 0.131 | 0.649 | 0.35-1.19 | 0.162 |
| *Comorbid conditions* | | | | | | | | | |
| Hypertension | 0.572 | 0.33-1.01 | 0.053 | 0.251 | 0.09-0.70 | 0.008 | 0.938 | 0.47-1.86 | 0.854 |
| Heart failure | 0.336 | 0.15-0.77 | 0.009 | 0.000 | 0.00--- | 0.999 | 0.324 | 0.13-0.79 | 0.013 |
| LVEF <50% | 0.702 | 0.38-1.30 | 0.261 | 0.000 | 0.00---- | 0.999 | 0.520 | 0.27-1.02 | 0.058 |
| *Ischemic heart disease* | 0.528 | 0.29-0.96 | 0.036 | 0.598 | 0.16-2.26 | 0.449 | 0.381 | 0.19-0.76 | 0.006 |
| Recent ACS | 0.000 | 0.00--- | 0.999 | 0.000 | 0.00--- | 1.000 | 0.000 | 0.00--- | 1.000 |
| Prior MI | 0.979 | 0.46-2.11 | 0.956 | 1.271 | 0.11-14.47 | 0.847 | 0.639 | 0.28-1.46 | 0.288 |
| Chronic stable CAD | 0.386 | 0.12-1.22 | 0.105 | 0.000 | 0.00---- | 0.999 | 0.618 | 0.16-2.37 | 0.483 |
| PCI/Balloon angioplasty | 0.575 | 0.26-1.27 | 0.171 | 0.491 | 0.06-4.36 | 0.524 | 0.429 | 0.18-1.03 | 0.059 |
| CABG | 0.718 | 0.17-3.06 | 0.654 | ---- | ----- | ---- | 0.459 | 0.11-1.98 | 0.296 |
| Cardiomyopathy | 0.787 | 0.37-1.69 | 0.540 | 1.012 | 0.19-5.47 | 0.989 | 0.570 | 0.24-1.37 | 0.208 |
| Valvular disease | 0.944 | 0.42-2.15 | 0.891 | 1.620 | 0.54-4.84 | 0.388 | 0.986 | 0.26-3.79 | 0.984 |
| Supraventricular arrhythmias (SA+AFL) | 0.865 | 0.48-1.57 | 0.635 | 1.317 | 0.45-3.82 | 0.613 | 0.653 | 0.31-1.37 | 0.258 |
| Ventricular arrhythmias | 0.575 | 0.26-1.27 | 0.171 | 0.706 | 0.14-3.57 | 0.674 | 0.447 | 0.18-1.13 | 0.089 |
| *CIEDs* | 0.658 | 0.28-1.54 | 0.333 | 0.296 | 0.04-2.46 | 0.260 | 0.774 | 0.28-2.15 | 0.622 |
| Anti-bradycardia pacemaker | 0.871 | 0.34-2.22 | 0.772 | 0.405 | 0.05-3.49 | 0.410 | 1.112 | 0.34-3.63 | 0.860 |
| ICD | 0.000 | 0.00---- | 0.999 | 0.000 | 0.00---- | 1.000 | 0.000 | 0.00--- | 1.000 |
| CRT | 0.398 | 0.04-3.87 | 0.427 | 0.000 | 0.00--- | 1.000 | 0.389 | 0.04-4.36 | 0.444 |
| Peripheral artery disease | 0.000 | 0.00---- | 0.999 | 0.000 | 0.00---- | 0.999 | 0.000 | 0.00---- | 0.999 |
| Diabetes mellitus type II | 0.522 | 0.29-0.93 | 0.027 | 0.370 | 0.12-1.16 | 0.088 | 0.613 | 0.30-1.26 | 0.181 |
| Prior stroke/TIA | 1.428 | 0.47-4.34 | 0.530 | 1.977 | 0.42-9.31 | 0.389 | 1.600 | 0.29-8.94 | 0.592 |
| Chronic kidney disease | 0.544 | 0.24-1.24 | 0.148 | 0.343 | 0.04-2.89 | 0.325 | 0.491 | 0.19-1.26 | 0.138 |
| COPD | 0.294 | 0.11-0.81 | 0.018 | 0.296 | 0.04-2.46 | 0.260 | 0.261 | 0.08-0.85 | 0.026 |
| Malignancy | 0.243 | 0.07-0.86 | 0.029 | 0.405 | 0.05-3.49 | 0.410 | 0.183 | 0.04-0.89 | 0.035 |
| Thyroid dysfunction | 0.760 | 0.44-1.30 | 0.315 | 0.603 | 0.24-1.55 | 0.293 | 1.044 | 0.51-2.14 | 0.906 |
| Hyperlipoproteinemia | 0.80 | 0.52-1.30 | 0.395 | 0.968 | 0.43-2.19 | 0.937 | 0.725 | 0.41-1.29 | 0.277 |
| Other conditions | 0.335 | 0.15-0.73 | 0.006 | 0.188 | 0.03-1.50 | 0.115 | 0.324 | 0.13-0.79 | 0.013 |
| CHA_2_DS_2_-VASc score | 0.633 | 0.54-0.75 | <0.001 | 0.652 | 0.46-0.92 | 0.016 | 0.712 | 0.58-0.87 | 0.001 |
| >1 non-sex CHA_2_DS_2_-VASc risk factors | 0.490 | 0.23-1.05 | 0.065 | 0.375 | 0.07-1.95 | 0.244 | 0.681 | 0.29-1.62 | 0.385 |
| *Current medication* | | | | | | | | | |
| OAC | 0.615 | 0.30-1.28 | 0.194 | 0.988 | 0.29-3.38 | 0.984 | 0.332 | 0.11-1.05 | 0.060 |
| VKA | 0.505 | 0.33-0.79 | 0.002 | 0.877 | 0.40-1.90 | 0.739 | 0.316 | 0.17-0.57 | <0.001 |
| NOAC | 1.747 | 1.10-2.77 | 0.048 | 1.149 | 0.51-2.58 | 0.736 | 2.455 | 1.32-4.57 | 0.005 |
| OAC treatment duration (years) | 1.009 | 0.95-1.07 | 0.775 | 1.086 | 0.97-1.22 | 0.173 | 0.971 | 0.90-1.04 | 0.416 |
| ASA | 0.518 | 0.25-1.10 | 0.085 | 0.171 | 0.02-1.36 | 0.095 | 0.691 | 0.28-1.71 | 0.423 |
| P2Y_12_ inhibitor | 0.249 | 0.09-0.68 | 0.006 | 0.000 | 0.00--- | 0.999 | 0.249 | 0.09-0.72 | 0.010 |
| Beta blocker | 1.925 | 1.10-3.38 | 0.023 | 0.986 | 0.39-2.50 | 0.976 | 2.747 | 1.33-5.67 | 0.006 |
| Non-DHP Ca blocker | 0.167 | 0.02-1.37 | 0.096 | 0.000 | 0.00--- | 0.999 | 0.190 | 0.02-1.73 | 0.141 |
| Digitalis | 0.468 | 0.14-1.53 | 0.208 | 0.621 | 0.07-5.76 | 0.675 | 0.378 | 0.09-1.56 | 0.178 |
| Antiarrhythmic drugs | 0.933 | 0.60-1.45 | 0.759 | 0.711 | 0.33-1.56 | 0.394 | 1.140 | 0.65-1.99 | 0.645 |
| Mexiletine | ---- | ---- | ---- | ---- | ---- | ---- | ---- | ---- | ---- |
| Propafenone | 2.050 | 1.01-4.16 | 0.047 | 4.722 | 1.54-14.47 | 0.007 | 1.319 | 0.52-3.34 | 0.558 |
| Flecainide | 1.330 | 0.61-2.93 | 0.478 | 1.564 | 0.35-6.91 | 0.556 | 1.095 | 0.42-2.85 | 0.853 |
| Sotalol | 0.601 | 0.05-6.69 | 0.679 | 0.000 | 0.00---- | 0.999 | ---- | --- | 1.000 |
| Amiodarone | 0.646 | 0.41-1.01 | 0.056 | 0.292 | 0.12-0.71 | 0.007 | 0.961 | 0.54-1.70 | 0.891 |
| DHP Ca blocker | 0.563 | 0.35-0.90 | 0.016 | 0.686 | 0.31-1.53 | 0.359 | 0.621 | 0.34-1.15 | 0.129 |
| ACEI/ARB | 0.787 | 0.49-1.26 | 0.319 | 0.629 | 0.28-1.41 | 0.261 | 0.821 | 0.44-1.52 | 0.528 |
| Diuretic | 0.602 | 0.39-0.93 | 0.023 | 0.636 | 0.29-1.39 | 0.256 | 0.614 | 0.35-1.08 | 0.089 |
| Spironolactone | 0.595 | 0.35-0.998 | 0.049 | 0.285 | 0.08-1.02 | 0.054 | 0.591 | 0.32-1.10 | 0.096 |
| Statins | 0.809 | 0.52-1.26 | 0.348 | 1.333 | 0.62-2.89 | 0.466 | 0.680 | 0.39-1.20 | 0.184 |
| Sedative | 0.686 | 0.33-1.41 | 0.306 | 1.139 | 0.33-3.96 | 0.838 | 0.508 | 0.21-1.25 | 0.140 |
| PPI | 0.738 | 0.46-1.20 | 0.218 | 0.637 | 0.25-1.64 | 0.350 | 0.677 | 0.37-1.23 | 0.200 |
| Insulin | 0.209 | 0.05-0.96 | 0.044 | 0.343 | 0.04-2.89 | 0.325 | 0.190 | 0.02-1.73 | 0.141 |
| Oral antidiabetic drug | 0.635 | 0.34-1.18 | 0.153 | 0.344 | 0.10-1.25 | 0.104 | 0.897 | 0.40-2.00 | 0.791 |
| Other medications | 0.331 | 0.21-0.53 | <0.001 | 0.267 | 0.11-0.65 | 0.004 | 0.373 | 0.21-0.67 | 0.001 |
| *Non-pharmacological treatment* | | | | | | | | | |
| Ablation/ECV | 2.181 | 1.40-3.41 | 0.001 | 1.680 | 0.75-3.79 | 0.211 | 2.035 | 1.16-3.58 | 0.014 |
| ECV AF | 2.523 | 1.55-4.11 | <0.001 | 2.640 | 1.05-6.63 | 0.039 | 2.000 | 1.10-3.63 | 0.023 |
| ECV AFL | 0.802 | 0.13-4.86 | 0.810 | 0.000 | 0.00---- | 0.999 | 1.589 | 0.14-17.81 | 0.707 |
| AF Ablation | 1.704 | 0.95-3.06 | 0.073 | 1.317 | 0.45-3.82 | 0.613 | 1.826 | 0.86-3.88 | 0.117 |
| AFL Ablation | 1.526 | 0.40-5.79 | 0.534 | ---- | ---- | ---- | 0.986 | 0.26-3.79 | 0.984 |
| Ablation other arrhythmias | ----- | ---- | 0.999 | ----- | ---- | 1.000 | ----- | ---- | 1.000 |
| *Multimorbidity and polypharmacy* | | | | | | | | | |
| Polypharmacy | 0.446 | 0.27-0.73 | 0.001 | 0.361 | 0.15-0.85 | 0.019 | 0.522 | 0.28-0.97 | 0.039 |
| N of drugs | 0.793 | 0.72-0.87 | <0.001 | 0.735 | 0.61-0.89 | 0.001 | 0.813 | 0.73-0.91 | <0.001 |
| N of pills | 0.824 | 0.76-0.89 | <0.001 | 0.771 | 0.66-0.91 | 0.002 | 0.840 | 0.77-0.92 | <0.001 |
| N of drugs without OAC | 0.790 | 0.72-0.87 | <0.001 | 0.723 | 0.59-0.88 | 0.001 | 0.813 | 0.73-0.91 | <0.001 |
| N of pills without OAC | 0.821 | 0.76-0.89 | <0.001 | 0.764 | 0.65-0.90 | 0.001 | 0.840 | 0.77-0.92 | <0.001 |
| Parenteral drug use | 0.264 | 0.07-0.94 | 0.040 | 0.260 | 0.03-2.13 | 0.210 | 0.384 | 0.07-2.15 | 0.275 |
| N of parenteral applications daily | 0.602 | 0.36-1.01 | 0.056 | 0.552 | 0.22-1.41 | 0.213 | 0.741 | 0.38-1.45 | 0.381 |
| N of comorbidities | 0.720 | 0.62-0.83 | <0.001 | 0.689 | 0.51-0.93 | 0.014 | 0.709 | 0.60-0.85 | <0.001 |
| Patients with multimorbidity (without SA/ VA) | 0.391 | 0.15-0.995 | 0.049 | 0.247 | 0.04-1.55 | 0.135 | 0.551 | 0.18-1.65 | 0.286 |
| ***Treatment burden*** | | | | | | | | | |
| Total TB | 0.976 | 0.97-0.99 | <0.001 | 0.977 | 0.96-0.995 | 0.011 | 0.982 | 0.97-0.995 | 0.008 |
| TB ≥59 | 0.411 | 0.25-0.69 | 0.001 | 0.536 | 0.23-1.24 | 0.146 | 0.456 | 0.23-0.91 | 0.026 |
| TB ≤26 | 1.882 | 1.11-3.18 | 0.018 | 1.328 | 0.49-3.62 | 0.580 | 1.902 | 0.98-3.69 | 0.057 |
| ***Questions about OAC-related treatment burden*** | | | | | | | | | |
| Q1 | 0.960 | 0.81-1.14 | 0.638 | 0.923 | 0.70-1.22 | 0.575 | 1.057 | 0.82-1.36 | 0.672 |
| Q2 | 0.962 | 0.86-1.07 | 0.495 | 0.940 | 0.75-1.17 | 0.583 | 0.960 | 0.84-1.10 | 0.548 |
| Q3 | 0.957 | 0.86-1.07 | 0.442 | 0.915 | 0.74-1.13 | 0.414 | 0.995 | 0.86-1.15 | 0.940 |
| Q4 | 0.928 | 0.82-1.05 | 0.216 | 0.949 | 0.78-1.16 | 0.613 | 0.926 | 0.79-1.08 | 0.330 |
| ***Questions about other drugs-related treatment burden*** | | | | | | | | | |
| Q1 | 0.922 | 0.83-1.03 | 0.133 | 0.969 | 0.82-1.15 | 0.722 | 0.914 | 0.80-1.05 | 0.203 |
| Q2 | 0.876 | 0.79-0.97 | 0.010 | 0.867 | 0.73-1.03 | 0.101 | 0.916 | 0.80-1.05 | 0.199 |
| Q3 | 0.935 | 0.85-1.03 | 0.159 | 0.942 | 0.80-1.10 | 0.462 | 0.956 | 0.85-1.08 | 0.477 |
| Q4 | 0.906 | 0.82-0.998 | 0.046 | 0.908 | 0.77-1.07 | 0.246 | 0.935 | 0.82-1.06 | 0.309 |
| ***Questions about other aspects of treatment burden*** | | | | | | | | | |
| Q1 | 0.878 | 0.81-0.95 | 0.001 | 0.873 | 0.76-1.00 | 0.051 | 0.894 | 0.81-0.99 | 0.027 |
| Q2 | 0.912 | 0.85-0.98 | 0.016 | 0.889 | 0.78-1.01 | 0.074 | 0.954 | 0.86-1.06 | 0.362 |
| Q3 | 0.881 | 0.82-0.94 | <0.001 | 0.860 | 0.76-0.97 | 0.017 | 0.917 | 0.84-1.00 | 0.050 |
| Q4 | 0.976 | 0.92-1.03 | 0.400 | 0.949 | 0.86-1.05 | 0.310 | 1.004 | 0.94-1.08 | 0.907 |
| Q5 | 0.944 | 0.89-1.00 | 0.051 | 0.978 | 0.89-1.08 | 0.654 | 0.941 | 0.87-1.02 | 0.118 |
| Q6 | 0.879 | 0.82-0.95 | 0.001 | 0.891 | 0.79-1.01 | 0.061 | 0.922 | 0.84-1.02 | 0.113 |
| Q7 | 0.755 | 0.69-0.83 | <0.001 | 0.776 | 0.66-0.91 | 0.002 | 0.759 | 0.67-0.86 | <0.001 |
| Q8 | 0.775 | 0.67-0.90 | 0.001 | 0.878 | 0.73-1.06 | 0.170 | 0.712 | 0.55-0.92 | 0.009 |
| Q9 | 0.867 | 0.80-0.94 | <0.001 | 0.906 | 0.80-1.02 | 0.115 | 0.875 | 0.79-0.97 | 0.013 |

**AF:** Atrial fibrillation; **LVEF:** Left ventricular ejection fraction; **ACS:** Acute coronary syndrome; **MI:** Myocardial infarction; **CAD:** Coronary artery disease; **PCI:** Percutaneous coronary intervention; **CABG:** Coronary artery bypass grafting; **AFL:** Atrial flutter; **CIED:** Cardiac implantable electronic devices; **ICD:** Implantable cardioverter defibrillator; **CRT:** Cardiac resynchronisation therapy; **TIA:** Transient ischemic attack, **COPD:** Chronic obstructive pulmonary disease; **OAC:** Oral anticoagulant therapy; **VKA:** Vitamin K antagonist; **NOAC:** Non-vitamin K antagonist oral anticoagulant; **ASA:** Acetylsalicylic acid; **DHP:** Dihydropyridine; **ACEI:** Angiotensin-converting enzyme inhibitor; **ARB:** Angiotensin receptor inhibitor; **PPI:** Proton pump inhibitor; **ECV:** electrical cardioversion; **SA:** Supraventricular arrhythmias; **VA:** Ventricular arrhythmias; **N:** Number; **Q:** Question.

**Table S9.** **Multivariable linear and logistic regression analyses of QOL 5D in AF patients.**

| **Multivariable Linear Regression analysis** | | | | |
| --- | --- | --- | --- | --- |
|  | **Variable** | **Beta** | **95% CI** | **P value** |
| **Female** | Age | 0.156 | 0.01-0.11 | 0.032 |
|  | Mobile with help | 0.149 | 0.06-3.73 | 0.043 |
|  | Hypertension | 0.168 | 0.22-2.93 | 0.023 |
|  | ICD | 0.230 | 3.36-13.61 | 0.001 |
|  | Non-DHP Ca blocker | 0.251 | 2.47-8.33 | <0.001 |
|  | Q7 about other aspects of TB | 0.237 | 0.13-0.41 | <0.001 |
|  | TB ≥59 | 0.182 | 0.25-2.20 | 0.015 |
|  | | | | |
| **Male** | Non-smoker | -0.115 | -1.36-(-0.06) | 0.032 |
|  | N of comorbidities | 0.160 | 0.09-0.473 | 0.004 |
|  | Sedative | 0.208 | 1.05-3.08 | <0.001 |
|  | Peripheral artery disease | 0.297 | 6.04-12.53 | <0.001 |
|  | Q7 about other aspects of TB | 0.297 | 0.22-0.45 | <0.001 |
|  | Q8 about other aspects of TB | 0.186 | 0.16-0.56 | 0.001 |
|  | Mobile with help | 0.234 | 1.93-5.08 | <0.001 |
| **Multivariable Logistic Regression analysis of the highest QOL quartile (≥6)** | | | | |
|  | **Variable** | **OR** | **95% CI** | **P value** |
| **Female** | Widow | 3.300 | 1.08-10.06 | 0.036 |
|  | Hyperlipoproteinemia | 3.163 | 1.17-8.56 | 0.023 |
|  | Polypharmacy | 4.468 | 1.03-19.34 | 0.045 |
|  | Q3 about other aspects of TB | 1.169 | 1.02-1.34 | 0.022 |
|  | Q7 about other aspects of TB | 1.199 | 1.04-1.38 | 0.012 |
|  | Fully mobile | 0.046 | 0.00-0.50 | 0.012 |
|  | | | | |
| **Male** | Former smoker | 4.306 | 1.20-15.41 | 0.025 |
|  | Heart failure | 15.927 | 4.00-63.47 | <0.001 |
|  | Sedative | 7.833 | 1.87-32.80 | 0.005 |
|  | Q7 about other aspects of TB | 1.308 | 1.07-1.60 | 0.008 |
|  | Fully mobile | 0.047 | 0.00-0.41 | 0.005 |
| **Multivariable Logistic Regression analysis of the lowest QOL quartile (≤1)** | | | | |
|  | **Variable** | **OR** | **95% CI** | **P value** |
| **Female** | Propafenone | 4.419 | 1.17-16.71 | 0.028 |
|  | N of drugs without OAC | 0.663 | 0.52-0.84 | 0.001 |
|  | Total TB | 0.978 | 0.96-1.00 | 0.050 |
|  | Q7 about other aspects of TB | 0.767 | 0.63-0.93 | 0.006 |
|  | | | | |
| **Male** | Beta blocker | 2.428 | 1.06-5.56 | 0.036 |
|  | Widower | 0.161 | 0.04-0.73 | 0.018 |
|  | N of comorbidities | 0.816 | 0.67-0.994 | 0.043 |
|  | VKA | 0.385 | 0.19-0.78 | 0.008 |
|  | Q7 about other aspects of TB | 0.773 | 0.68-0.88 | <0.001 |
|  | Q8 about other aspects of TB | 0.752 | 0.58-0.98 | 0.036 |

**ICD:** Implantable cardioverter defibrillator; **CAD:** Coronary artery disease; **N:** Number; **Q:** Question; **TB:**  Treatment burden; **DHP:** Dihydropyridine; **VKA:** Vitamin K antagonist**; ECV:** electrical cardioversion; **AF:** Atrial fibrillation; **PPI:** Proton pump inhibitor; **PCI:** Percutaneous coronary intervention; **NOAC:** Non-vitamin k antagonist oral anticoagulant.

Table S10. Relations of self-estimated health status rating with treatment burden in AF cohort, females and males.

| **Self-estimated health status rating as a continuous variable** | | | | | | | | | |
| --- | --- | --- | --- | --- | --- | --- | --- | --- | --- |
| **Variable** | **AF patients**  **n=331 (%)** | | | **Female**  **n=127 (38.4)** | | | **Male**  **n=204 (61.6)** | | |
|  | **beta** | **CI 95%** | **P value** | **beta** | **CI 95%** | **P value** | **beta** | **CI 95%** | **P value** |
| ***Treatment burden*** | | | | | | | | | |
| Total TB | -0.200 | -0.25-(-0.08) | <0.001 | -0.116 | -0.22-0.05 | 0.195 | -0.224 | -0.33-(-0.08) | 0.001 |
| TB ≥59 | -0.190 | -13.55-(-3.80) | 0.001 | -0.143 | -12.88-1.32 | 0.110 | -0.193 | -16.71-(-2.87) | 0.006 |
| TB ≤26 | 0.124 | 0.80-11.37 | 0.024 | 0.119 | -3.01-15.58 | 0.183 | 0.109 | -1.35-11.66 | 0.120 |
| ***Questions about OAC-related treatment burden*** | | | | | | | | | |
| Q1 | -0.070 | -2.80-0.59 | 0.201 | -0.081 | -3.28-1.21 | 0.364 | -0.047 | -3.43-1.69 | 0.502 |
| Q2 | -0.038 | -1.49-0.72 | 0.489 | -0.037 | -2.20-1.43 | 0.676 | -0.043 | -1.82-0.96 | 0.543 |
| Q3 | -0.084 | -2.00-0.25 | 0.127 | -0.101 | -2.65-0.72 | 0.258 | -0.065 | -2.17-0.78 | 0.357 |
| Q4 | -0.041 | -1.60-0.72 | 0.456 | 0.029 | -1.41-1.96 | 0.748 | -0.080 | -2.49-0.66 | 0.253 |
| ***Questions about other drugs-related treatment burden*** | | | | | | | | | |
| Q1 | -0.093 | -1.91-0.14 | 0.090 | -0.090 | -2.24-0.72 | 0.313 | -0.082 | -2.27-0.58 | 0.242 |
| Q2 | -0.059 | -1.44-0.43 | 0.287 | 0.024 | -1.10-1.44 | 0.792 | -0.096 | -2.33-0.42 | 0.173 |
| Q3 | -0.055 | -1.39-0.46 | 0.321 | 0.00 | -1.34-1.34 | 0.997 | -0.075 | -1.97-0.59 | 0.289 |
| Q4 | -0.058 | -1.42-0.43 | 0.291 | -0040 | -1.57-0.98 | 0.652 | -0.049 | -1.80-0.87 | 0.488 |
| ***Questions about other aspects of treatment burden*** | | | | | | | | | |
| Q1 | -0.166 | -1.83-(-0.40) | 0.002 | -0.063 | -1.43-0.68 | 0.482 | -0.220 | -2.57-(-0.61) | 0.002 |
| Q2 | -0.202 | -2.09-(-0.65) | <0.001 | -0.158 | -1.94-0.10 | 0.077 | -0.216 | -2.67-(-0.61) | 0.002 |
| Q3 | -0.159 | -1.62-(-0.32) | 0.004 | -0.075 | -1.43-0.58 | 0.401 | -0.185 | -2.05-(-0.31) | 0.008 |
| Q4 | -0.072 | -0.95-0.19 | 0.191 | -0.019 | -1.01-0.81 | 0.835 | -0.090 | -1.22-0.26 | 0.200 |
| Q5 | -0.025 | -0.72-0.45 | 0.649 | -0.063 | -1.20-0.57 | 0.480 | 0.021 | -0.67-0.91 | 0.769 |
| Q6 | -0.206 | -2.02-(-0.64) | <0.001 | -0.304 | -2.70-(-0.77) | 0.001 | -0.094 | -1.72-0.33 | 0.181 |
| Q7 | -0.234 | -2.28-(-0.86) | <0.001 | -0.145 | -1.89-0.18 | 0.103 | -0.272 | -3.02-(-1.03) | <0.001 |
| Q8 | -0.095 | -2.00-0.12 | 0.083 | 0.147 | -0.21-2.44 | 0.098 | -0.299 | -5.59-(-2.15) | <0.001 |
| Q9 | -0.108 | -1.44-(-0.001) | 0.050 | 0.002 | -1.01-1.03 | 0.983 | -0.154 | -2.20-(-0.13) | 0.028 |
| **The lowest self-estimated health status rating quartile (≤50 points)** | | | | | | | | | |
| **Variable** | **AF patients**  **n=124 (%)** | | | **Female**  **n=57 (44.9)** | | | **Male**  **n=67 (32.8)** | | |
|  | **OR** | **CI 95%** | **P value** | **OR** | **CI 95%** | **P value** | **OR** | **CI 95%** | **P value** |
| ***Treatment burden*** | | | | | | | | | |
| Total TB | 1.013 | 1.00-1.02 | 0.006 | 1.008 | 0.995-1.02 | 0.245 | 1.015 | 1.00-1.03 | 0.030 |
| TB ≥59 | 2.185 | 1.33-3.58 | 0.002 | 2.100 | 1.01-4.35 | 0.046 | 1.962 | 0.98-3.93 | 0.057 |
| TB ≤26 | 0.715 | 0.41-1.24 | 0.235 | 0.716 | 0.27-1.87 | 0.495 | 0.780 | 0.39-1.55 | 0.478 |
| ***Questions about OAC-related treatment burden*** | | | | | | | | | |
| Q1 | 1.112 | 0.94-1.32 | 0.214 | 1.111 | 0.88-1.40 | 0.372 | 1.082 | 0.84-1.39 | 0.538 |
| Q2 | 1.058 | 0.95-1.18 | 0.310 | 1.103 | 0.92-1.33 | 0.302 | 1.039 | 0.91-1.19 | 0.587 |
| Q3 | 1.051 | 0.94-1.17 | 0.373 | 1.041 | 0.88-1.23 | 0.640 | 1.049 | 0.91-1.21 | 0.519 |
| Q4 | 1.016 | 0.91-1.14 | 0.785 | 0.948 | 0.80-1.13 | 0.546 | 1.067 | 0.91-1.25 | 0.411 |
| ***Questions about other drugs-related treatment burden*** | | | | | | | | | |
| Q1 | 1.106 | 0.999-1.22 | 0.053 | 1.054 | 0.91-1.22 | 0.489 | 1.138 | 0.99-1.31 | 0.070 |
| Q2 | 1.069 | 0.98-1.17 | 0.155 | 1.032 | 0.91-1.17 | 0.623 | 1.083 | 0.95-1.24 | 0.253 |
| Q3 | 1.047 | 0.96-1.15 | 0.332 | 0.979 | 0.86-1.12 | 0.758 | 1.092 | 0.96-1.24 | 0.170 |
| Q4 | 1.078 | 0.98-1.18 | 0.105 | 1.064 | 0.94-1.21 | 0.341 | 1.070 | 0.94-1.22 | 0.312 |
| ***Questions about other aspects of treatment burden*** | | | | | | | | | |
| Q1 | 1.113 | 1.04-1.20 | 0.004 | 1.101 | 0.99-1.23 | 0.082 | 1.111 | 1.01-1.23 | 0.039 |
| Q2 | 1.165 | 1.08-1.26 | <0.001 | 1.168 | 1.05-1.30 | 0.005 | 1.146 | 1.03-1.27 | 0.011 |
| Q3 | 1.047 | 0.98-1.12 | 0.175 | 0.995 | 0.90-1.10 | 0.930 | 1.068 | 0.98-1.17 | 0.149 |
| Q4 | 1.026 | 0.97-1.09 | 0.382 | 1.013 | 0.92-1.11 | 0.784 | 1.028 | 0.95-1.11 | 0.484 |
| Q5 | 0.988 | 0.93-1.05 | 0.702 | 0.987 | 0.90-1.08 | 0.776 | 0.976 | 0.90-1.06 | 0.562 |
| Q6 | 1.120 | 1.04-1.20 | 0.002 | 1.180 | 1.06-1.31 | 0.003 | 1.039 | 0.94-1.15 | 0.471 |
| Q7 | 1.130 | 1.05-1.22 | 0.001 | 1.081 | 0.97-1.20 | 0.151 | 1.156 | 1.04-1.28 | 0.006 |
| Q8 | 1.003 | 0.90-1.12 | 0.957 | 0.852 | 0.73-0.998 | 0.047 | 1.221 | 1.02-1.47 | 0.035 |
| Q9 | 1.024 | 0.95-1.10 | 0.516 | 0.969 | 0.87-1.07 | 0.544 | 1.053 | 0.95-1.17 | 0.333 |
| **The highest self-estimated health status rating quartile (≥75 points)** | | | | | | | | | |
| **Variable** | **AF patients**  **n=98 (%)** | | | **Female**  **n=27 (21.3)** | | | **Male**  **n=71 (34.8)** | | |
|  | **OR** | **CI 95%** | **P value** | **OR** | **CI 95%** | **P value** | **OR** | **CI 95%** | **P value** |
| ***Treatment burden*** | | | | | | | | | |
| Total TB | 0.983 | 0.97-0.99 | 0.001 | 0.994 | 0.98-1.01 | 0.482 | 0.975 | 0.96-0.99 | 0.002 |
| TB ≥59 | 0.419 | 0.23-0.77 | 0.005 | 0.632 | 0.25-1.58 | 0.326 | 0.370 | 0.16-0.85 | 0.019 |
| TB ≤26 | 1.671 | 0.97-2.88 | 0.065 | 0.849 | 0.26-2.77 | 0.786 | 1.915 | 1.01-3.65 | 0.048 |
| ***Questions about OAC-related treatment burden*** | | | | | | | | | |
| Q1 | 0.823 | 0.65-1.05 | 0.116 | 0.892 | 0.64-1.25 | 0.504 | 0.787 | 0.55-1.12 | 0.183 |
| Q2 | 0.960 | 0.85-1.09 | 0.521 | 0.990 | 0.79-1.24 | 0.930 | 0.943 | 0.81-1.10 | 0.442 |
| Q3 | 0.710 | 0.57-0.89 | 0.003 | 0.315 | 0.07-1.38 | 0.125 | 0.790 | 0.63-0.99 | 0.038 |
| Q4 | 0.941 | 0.82-1.08 | 0.372 | 0.944 | 0.75-1.19 | 0.620 | 0.948 | 0.80-1.12 | 0.533 |
| ***Questions about other drugs-related treatment burden*** | | | | | | | | | |
| Q1 | 0.892 | 0.78-101 | 0.081 | 0.949 | 0.78-1.16 | 0.600 | 0.869 | 0.73-1.03 | 0.105 |
| Q2 | 0.961 | 0.87-1.07 | 0.455 | 1.062 | 0.92-1.23 | 0.421 | 0.905 | 0.78-1.06 | 0.209 |
| Q3 | 0.887 | 0.79-0.997 | 0.044 | 0.975 | 0.82-1.16 | 0.767 | 0.841 | 0.71-0.99 | 0.039 |
| Q4 | 0.916 | 0.82-1.02 | 0.123 | 0.917 | 0.76-1.10 | 0.347 | 0.936 | 0.81-1.08 | 0.371 |
| ***Questions about other aspects of treatment burden*** | | | | | | | | | |
| Q1 | 0.895 | 0.82-0.98 | 0.013 | 1.000 | 0.88-1.14 | 0.997 | 0.837 | 0.74-0.95 | 0.005 |
| Q2 | 0.861 | 0.78-0.95 | 0.002 | 0.903 | 0.78-1.04 | 0.361 | 0.845 | 0.74-0.96 | 0.009 |
| Q3 | 0.952 | 0.89-1.02 | 0.188 | 1.032 | 0.91-1.17 | 0.609 | 0.931 | 0.85-1.02 | 0.134 |
| Q4 | 0.972 | 0.91-1.03 | 0.360 | 1.005 | 0.90-1.12 | 0.924 | 0.965 | 0.90-1.04 | 0.346 |
| Q5 | 0.996 | 0.94-1.06 | 0.903 | 1.025 | 0.92-1.14 | 0.659 | 0.995 | 0.92-1.08 | 0.895 |
| Q6 | 0.842 | 0.77-0.92 | <0.001 | 0.829 | 0.72-0.96 | 0.012 | 0.875 | 0.78-0.96 | 0.008 |
| Q7 | 0.945 | 0.84-1.07 | 0.375 | 1.099 | 0.95-1.27 | 0.210 | 0.733 | 0.53-1.02 | 0.061 |
| Q8 | 0.909 | 0.84-0.99 | 0.028 | 1.010 | 0.89-1.14 | 0.871 | 0.856 | 0.76-0.97 | 0.015 |
| Q9 | 0.909 | 0.84-0.99 | 0.028 | 1.010 | 0.89-1.14 | 0.871 | 0.856 | 0.76-0.97 | 0.015 |

**AF:** Atrial fibrillation; **TB:** Treatment burden; **Q:** Question; **OAC:** Oral anticoagulants.
